# Control strategies for active lower extremity prosthetics and orthotics: a review

**DOI:** 10.1186/1743-0003-12-1

**Published:** 2015-01-05

**Authors:** Michael R Tucker, Jeremy Olivier, Anna Pagel, Hannes Bleuler, Mohamed Bouri, Olivier Lambercy, José del R Millán, Robert Riener, Heike Vallery, Roger Gassert

**Affiliations:** Rehabilitation Engineering Lab, Department of Health Sciences and Technology, ETH Zurich, Zürich, Switzerland; Robotic Systems Laboratory, Institute for Microengineering, EPFL, Lausanne, Switzerland; Sensory Motor Systems Lab, Department of Health Sciences and Technology, ETH Zurich, Zürich, Switzerland; Defitech Chair in Non-Invasive Brain-Machine Interface, Center for Neuroprosthetics, Institute of Bioengineering, EPFL, Lausanne, Switzerland; Faculty of Medicine, Spinal Cord Injury Center, Balgrist University Hospital, University of Zurich, Zürich, Switzerland; Faculty of Mechanical, Maritime and Materials Engineering, Department of BioMechanical Engineering, Delft University of Technology, Delft, The Netherlands

**Keywords:** Prosthetic, Orthotic, Exoskeleton, Control architecture, Intention recognition, Activity mode recognition, Volitional control, Shared control, Finite-state machine, Electromyography, Sensory feedback, Sensory substitution, Seamless integration, Sensory-motor control, Rehabilitation robotics, Bionic, Biomechatronic, Legged locomotion

## Abstract

Technological advancements have led to the development of numerous wearable robotic devices for the physical assistance and restoration of human locomotion. While many challenges remain with respect to the mechanical design of such devices, it is at least equally challenging and important to develop strategies to control them in concert with the intentions of the user.

This work reviews the state-of-the-art techniques for controlling portable active lower limb prosthetic and orthotic (P/O) devices in the context of locomotive activities of daily living (ADL), and considers how these can be interfaced with the user’s sensory-motor control system. This review underscores the practical challenges and opportunities associated with P/O control, which can be used to accelerate future developments in this field. Furthermore, this work provides a classification scheme for the comparison of the various control strategies.

As a novel contribution, a general framework for the control of portable gait-assistance devices is proposed. This framework accounts for the physical and informatic interactions between the controller, the user, the environment, and the mechanical device itself. Such a treatment of P/Os – not as independent devices, but as actors within an ecosystem – is suggested to be necessary to structure the next generation of intelligent and multifunctional controllers.

Each element of the proposed framework is discussed with respect to the role that it plays in the assistance of locomotion, along with how its states can be sensed as inputs to the controller. The reviewed controllers are shown to fit within different levels of a hierarchical scheme, which loosely resembles the structure and functionality of the nominal human central nervous system (CNS). Active and passive safety mechanisms are considered to be central aspects underlying all of P/O design and control, and are shown to be critical for regulatory approval of such devices for real-world use.

The works discussed herein provide evidence that, while we are getting ever closer, significant challenges still exist for the development of controllers for portable powered P/O devices that can seamlessly integrate with the user’s neuromusculoskeletal system and are practical for use in locomotive ADL.

## Introduction

An exciting revolution is underway in the fields of rehabilitation and assistive robotics, where technologies are being developed to actively aid or restore legged locomotion to individuals suffering from muscular impairments or weakness, neurologic injury, or amputations affecting the lower limbs.

Examples of energetically passive prosthetic and orthotic (P/O) devices date back thousands of years and have been used with varying levels of success [1]. Owing to largely to their relative simplicity, low up-front cost and robust design, passive devices are a practical means to enable functional restoration of gait for many conditions. The inherent shortcomings of these devices are their inability to generate mechanical power, their failure to autonomously adapt to the user’s changing needs, and the lack of sensory feedback that they provide to the user regarding the states of the limb and of the device. Each of these aspects are required for seamless cognitive and physical interaction between the device and the user.

Intelligent and portable actuated P/Os have the potential to dramatically improve the mobility, and therefore quality of life, of people with locomotive impairments. As such devices begin to approach the power output, efficiency, and versatility of the limbs that they assist or replace, the end-users will be (re)enabled to partake in activities of daily living (ADLs) that require net-positive energetic output (e.g. stair climbing, running, jumping) in the same ways that an able-bodied counterpart would. Relative to their passive counterparts, active P/Os also have the potential to increase self-selected gait speed while reducing metabolic expenditure [2–4]. Such devices may also increase gait symmetry and reduce wear-and-tear on the user’s unaffected joints that could otherwise arise due to compensatory movements.

While the potential benefits that such devices may deliver are compelling on their own, the statistics regarding the populations who may benefit from them are also convincing arguments for their continued development. Given the projected demographic shift toward an older population [5], an increase in age-correlated conditions associated with pathological gait (e.g. stroke [6], spinal cord injury [7], Parkinson’s disease [8], and lower limb amputations [9]) can likewise be expected. Robotic P/O devices may provide more intensive and purposeful therapeutic training through ADLs, while also reducing the burdens placed on the short supply of therapists and other health care personnel.

Advancements in actuation, energy storage, miniaturized sensing, automated pattern recognition, and embedded computational technology have lead to the development of a number of mobile robotic devices for the assistance and restoration of human locomotion. Within the next decade it is expected that many more active lower limb prostheses, exoskeletons, and orthoses will be developed and commercialized.

While many engineering challenges remain with regard to the mechanical design of such devices, additional questions remain with respect to how these devices may be controlled in concert with the user’s remaining (impaired and unimpaired) sensory-motor control system. For example, how can the physical and cognitive interaction between the user and a powered lower limb P/O device be improved through various control strategies, beyond the state-of-the-art? How can the control approaches be generalized across different types of devices and the various joints that they actuate? How is locomotion nominally controlled in healthy humans, and how can this information be applied to the estimation of the user’s locomotive intent and to the structure of a P/O controller? What are the major challenges and opportunities that are likely to be encountered as these devices leave well-characterized research environments and enter the real world? Only once each of these aspects have been sufficiently addressed will it be possible for robotic assistive devices to demonstrate their efficacy and to become commonplace in real-world environments.

The objective of this review is to provide some answers to these questions based on our current understanding of the problems underlying the control of lower limb P/Os and the strategies that have been used to overcome them. As a novel contribution, we present a general framework for the classification and design of controllers for portable lower limb P/O devices. It promotes a common vocabulary and facilitates the cross-pollination of ideas between these very similar, yet fundamentally different, classes of devices. Furthermore, this review underscores the challenges associated with the seamless integration of a P/O device with the sensory-motor control system of the user. Through the referencing and classification of the state-of-the-art control strategies, this review is intended to provide guidelines for the acceleration of future developments, especially in the context of active physical P/O assistance with locomotive ADLs.

### Definitions, scope and prior work

Adopting the terminology provided by the review of Herr [10], the term *exoskeleton* is used to describe a device that enhances the physical capabilities of an able-bodied user, whereas the term *orthosis* is used to describe a device used to assist a person with an impairment of the limbs. Though exceptions exist, orthoses and exoskeletons typically act in parallel with the limb. A *prosthesis* is a device which supplants a missing limb, and therefore acts in series with the residual limb.

Several related review papers have been published in recent years that comprehensively establish the state-of-the-art in portable and active lower limb prosthetics, orthotics and exoskeletons, mostly in terms of the design and hardware realization [10–15]. While these reviews do touch on some of the implemented control strategies, the holistic descriptions of the considered devices often do not leave room to ruminate on this particular subject. Chapters 4 and 5 of [16] provide a nice depth of theory regarding cognitive and physical human-robot interaction, which complements the breadth of practical examples provided herein.

Controllers for robotic prosthetic, orthotic and exoskeletal systems for the ankle were recently reviewed by Jimenez-Fabian and Verlinden [17]. The present work extends their review by considering controllers for the hip, knee and ankle, with special emphasis on P/O devices. The discussion and classification of controllers herein is structured and enhanced by the provision of a generalized control framework. Furthermore, this architecture is also proposed as a template for the development of the next generation of multifunctional controllers for active lower limb P/O devices.

This review also considers modalities for artificial sensory substitution and feedback. Though much of the work in this field is relatively nascent in the context of robotic lower limb P/Os, this is seen as a promising and necessary future avenue of research for the seamless integration of the device’s controller with that of the human user.

It is duly noted that the power output characteristics vary substantially between the hip, knee, and ankle during a given activity [18]. Additionally, the nature of the physical assistance required of a prosthesis is substantially different than that of an orthosis for the corresponding joint. Though these differences fundamentally preclude the direct translation of control paradigms between devices, there are also many concepts that can be applied universally.

This review excludes explicit consideration of controllers for energetically net-passive devices and powered exoskeletons intended exclusively for performance augmentation of able-bodied users. Attention is only given to devices which are wearable and portable in nature, or in principle could be made as such in the near-future. This would exclude treadmill-based gait training orthoses such as the LOPES [19] and the Lokomat (Hocoma AG, Volketswil, Switzerland), which were among the classes of devices discussed in the review of Marchal-Crespo and Reinkensmeyer [20]. Furthermore, this excludes consideration of studies involving purely stimulatory devices that act in the absence of external mechanical assistance (e.g. functional electrical stimulation (FES)), which were reviewed in [21–23].

## Generalized control framework

**Figure 1:**
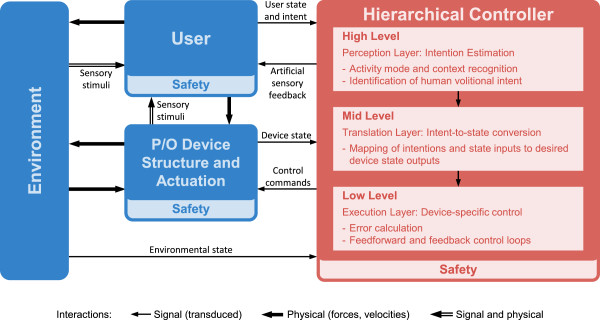
**Generalized control framework for active lower limb prostheses and orthoses.** The proposed framework illustrates the physical and signal-level interactions between a powered lower limb prosthetic or orthotic (P/O) device, a user, and his environment. The arrows indicate the exchange of power and information between the various components of the P/O ecosystem. A hierarchical control structure is implemented, with the estimation of the user’s locomotive intent taking place at the high level, translation of the user’s intent to a desired device state at the mid level, and a device-specific controller responsible for realizing the desired device state at the low level. Safety mechanisms underly all aspects of P/O design, including those which are mechanically passive and those which are actively controlled. Adapted from Varol et al. 2010 [24].

To structure the classification and discussion of the various control approaches for active lower limb P/Os, we propose the generalized framework of Figure 1. This framework was inspired by and extended from that of Varol et al. 2010 [24] to be applied to a wider range of devices (i.e. prostheses and orthoses) and joints (i.e. hip, knee and ankle). The diagram reflects the physical interaction and signal-level feedback loops underlying powered assistive devices during practical use. The major subsystems include a hierarchical control structure, the user of the P/O device, the environment through which he ambulates, and the device itself. The framework has been generalized to describe “what” each component of the hierarchical controller should do rather than “how” it should be done. Safety layers have been included to emphasize the importance of safe human-robot interaction, especially considering the amount of power such devices can generate. Furthermore, the structure of the rest of the paper follows that of this framework, which provides a holistic consideration of the challenges facing P/O control developments today.

Motion intentions originate with the user, whose physiological *state* and desires must be discerned and interpreted. In this context, the user’s *state* refers to the pose (i.e. position and orientation) and velocity of the head, trunk and limbs, as well as the existence and status of physical interactions between the user and the environment or the user and the P/O device.

Motion intention estimation requires an understanding of how locomotion is nominally controlled in humans and how the user’s state and intent can be sensed. The terrain features and surface conditions of the environment (i.e. the *environmental state*) constrain the type of movements that can be carried out, and if perceived by the controller can be taken into account. Interaction forces exist between the device, the user, and the environment, which can also be sensed as an input to the controller.

At the high level, the controller must perceive the user’s locomotive intent. Activity mode recognition identifies the current locomotive task, such as standing, level walking and stair descent. Direct volitional control allows the user to voluntarily manipulate the device’s state, i.e. joint positions, velocities and torques. It is possible to combine both of these, where the volitional control modulates the device’s behavior within a particular activity.

The mid-level controller translates the user’s motion intentions from the high level to desired device states for the low-level controller to track. It is at this level of control that the user’s state within the gait cycle is determined and a control law applied. It may have the form of a position/velocity, torque, impedance, or admittance controller.

The desired device state is passed to the low-level controller, which computes the error with respect to the current state. It then sends commands to the actuator(s) in an effort to reduce the error. This can be achieved through feedforward or feedback control, and typically accounts for the kinematic and kinetic properties of the device.

Finally, the P/O device is actuated to execute these commands, and thus the control loop is closed. The device may also provide artificial sensory feedback to the user for full integration with the physiological control system.

Given that a robotic P/O device is likely capable of generating substantial output forces and is to be placed in close physical contact with the user, both passive and active safety mechanisms are of paramount importance and must underly all aspects of device hardware and software design. Therefore, safety considerations are intended to be implicit to all subsystems of the generalized control architecture, despite the lack of explicit connections.

Each subsystem within the generalized control architecture can be defined by a set of physical and signal-level *inputs*, by a set of *processes* that operate on those inputs to control the exchange of power through the subsystem, and by a set of *outputs* that transmit power and signals to connected subsystems. In the following sections, each of these subsystems will be discussed with regard to the roles that they play in the proposed generalized control architecture for actively assisted locomotion with mobile lower limb P/O devices.

## The prosthesis/orthosis user

The overarching design goal for the controller of an assistive device is that of *seamless integration* with the user’s residual musculoskeletal system and sensory-motor control loops, all of which are under the supreme command of the central nervous system (CNS). In other words, the human and the robot must work together in an intuitive and synergistic way: the device recognizes the user’s motion intentions and acts to assist with that movement with minimal cognitive disruption and required compensatory motion, and rich sensory feedback is provided to the user. Thus, a well-designed and interactive P/O controller must begin with an understanding of the human controller.

First, the physiological systems responsible for the nominal control of locomotion in unaffected humans will be considered. This condition serves as a benchmark to contrast with the ensuing discussion on compensatory and assisted control of locomotion. Then, various portable sensor modalities that have been used in P/Os for the estimation of the user’s physical state and motion intentions are presented. Finally, techniques for providing artificial sensory feedback to the user regarding his interactions with the device and the environment are discussed.

### Nominal control of locomotion

Human control of locomotion is a fascinating area of ongoing research, where physiologists, neuroscientists and engineers are working to increase our understanding of the structure and functionality of nature’s most optimized controller, the CNS, and how it orchestrates movement.

**Figure 2:**
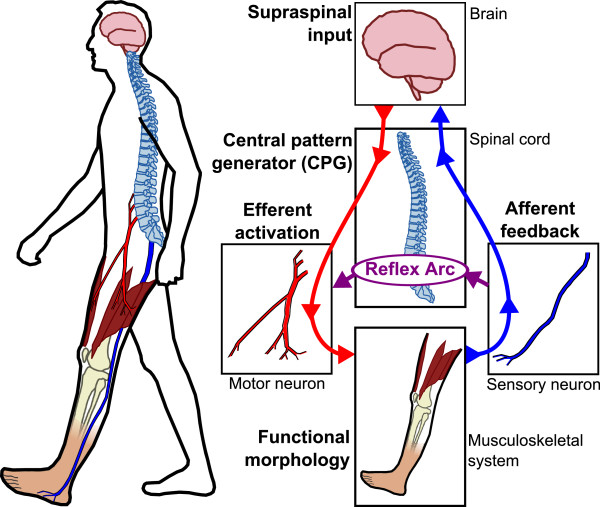
**Nominal sensory-motor control loop for human locomotion.** Motion intentions originate from supraspinal input, which along with afferent feedback serves to modulate basic underlying locomotor patterns within a network of spinal interneurons, commonly referred to as the central pattern generator (CPG). Efferent stimulation is transmitted through motor neurons to individual muscle groups, which are recruited to effect the movement. Afferent feedback, including that from proprioceptors of the muscles and joints and mechanoreceptors of the skin, is used to directly modulate motor commands via mono- and polysynaptic reflex arcs, thus contributing to the efficiency of gait under normal conditions and stability of gait in the face of unexpected perturbations. Sensory information is also transmitted to the brain, where it is combined with higher level inputs from the visual, auditory, and vestibular systems to provide information required for the maintenance of balance, orientation and control of precise movements.

It is widely accepted that human locomotion depends both on basic patterns generated at the spinal level, and the volitional and reflex-dependent fine control of these patterns at different levels [25–27] (Figure 2). Basic motor patterns are thought to be generated by a network of spinal interneurons, often referred to as the central pattern generator (CPG) [28–31].

The volitional control of movement and high-level modulation of locomotor patterns is originated at the supraspinal or cortical level, i.e. premotor and motor cortex, cerebellum and brain stem (Figure 2, top). The latter regulates both the CPG and reflex mechanisms [32]. Also at the supraspinal level, information from vestibular and visual systems are incorporated, which are crucial for the maintenance of balance, orientation, and control of precise movement [32].

Locomotor patterns are also modulated by afferent feedback arising from muscle spindles, Golgi tendon organs, mechanoreceptors lining the joint capsules, tactile mechanoreceptors and free nerve endings of the skin that sense stretch, pressure, heat, or pain [32, 33]. The modulation via reflexive pathways is twofold: taking place under normal conditions, principally to increase the efficiency of gait, and during unexpected perturbations, to stabilize posture [34, 35]. Following neurological injury, the reflexive behavior may be abnormal and can result, for example, in muscle spasticity.

Efferent nerve fibers, i.e. motor neurons, transmit the resulting motor commands to individual muscles, which are recruited to contract and thus to generate force about one or more joints of the skeletal system. Coordination of these forces through synergistic muscle activation and inter-joint coupling is exhibited during locomotor execution [31, 36]. Afferent nerve fibers, i.e. sensory neurons, transmit information from the musculoskeletal system to the CNS, thus closing the feedback loop for the nominal control of human locomotion.

Incidentally, some loose analogies can be made between the structure and functionality of the physiological sensory-motor control system of Figure 2 and the generalized control structure of Figure 1. For example, high-level motor commands and volitional control of movement originate at the supraspinal level of the human, which corresponds to the high level controller. These commands, along with afferent feedback via reflex arcs, modulate the basic patterns of the CPG. This is analogous to the integration of high-level commands with feedback from sensors in the mid level controller to determine a desired output behavior. The resulting motor commands are transmitted via motor neurons to the muscles, which then contract to generate movement about the joints. Proprioception provides feedback regarding the execution of movement. This is similar the action of the low level of the controller that sends commands to the actuators that move the structure of the P/O.

### Compensatory and assisted control of locomotion

In the wake of a neurologic injury or limb amputation, parts of the sensory-motor control loop responsible for locomotion may be disrupted and would need to be assisted or even taken over by a P/O device. Stemming from the inherent adaptability and plasticity of the CNS, compensatory mechanisms may arise to counteract the loss of structure and function post-disease or injury. These are typically manifested as a gait abnormality and may range from a simple limp to a total inability to walk, any of which may be considered to be the optimal outcome for a given condition [32]. Thus, the P/O controller must be robust enough to accommodate gait patterns that are potentially far-removed from the nominal condition.

Pathological gait has also been linked to numerous secondary conditions, including increased energy expenditure [37], increased risk and fear of falling [38, 39], and degenerative bone and joint disorders (e.g. osteoarthritis, osteopenia/osteoporosis, and back pain). These will not only involve the affected limb, but also the unaffected limb and others involved in compensatory movements [15, 40].

The purpose of a powered assistive device is to interface with the residual neuromusculoskeletal structures such that the support, control and actuation loops are reconnected. This provides the immediate benefit of re-enabling locomotive ADL, and potentially the long-term benefit of rehabilitating and retraining physiological gait patterns over time. This may result in a “spiral of adaptation” as the user adapts to the new conditions imposed by the use of a P/O device, and that the device itself may need to adapt to the evolving needs of the user [41].

Based on the review of Marchal-Crespo and Reinkensmeyer [20], most training paradigms for gait rehabilitation can be classified into two groups. An *assistive controller* directly helps the user in moving their affected limbs in accordance with the desired movement. A *challenge-based* controller could be used to provoke motor plasticity within the user by making movements more difficult through, for example, error amplification. While there remains some debate regarding which of these strategies would provide the most lasting rehabilitative benefit to the user when employed during a dedicated therapy session [42], intuition indicates that an assistive controller would provide the most utility in the performance of ADL in a real-world setting. This may at least partially explain why, within the scope of the devices covered in this review, no examples of challenge-based controllers were found.

It is left as an open question whether one of the control objectives of the device should be to minimize the user’s exhibition of compensatory mechanisms or whether restoration of functional ADLs is sufficient. In either case, an oft-cited hypothesis motivating the development of active P/Os is that only an actuated device would be capable of providing the full power-output capabilities of the corresponding physiological joints, and could thus enable gait patterns resembling those of unaffected persons across a wide variety of activities and terrain [15, 43]. The corollary is that the aforementioned secondary conditions could be prevented – providing a direct benefit for the user and a potential incentive for health care and insurance providers to opt for an active device as opposed to a passive one.

The take-away message is that a practical P/O controller must take into account the individual user’s capabilities and physiological constraints in order to realize functional outcomes. These can be achieved both through assistance and rehabilitation, either of which may dramatically improve the mobility and quality of life for the user.

### Sensor modalities for motion intention estimation

The intention of a user to execute a movement can be estimated through the sensing of cortical and neuromuscular activity, posture, locomotive state, and physical interaction with the environment and the P/O device. The sensor modalities corresponding to each of these differ widely in terms of their relative *invasiveness* and the *richness* of the provided information [15]. Here, invasiveness is intended to indicate the relative ease (in time, effort, and risk) with which a sensor may be applied and removed. These range from completely noninvasive (e.g. fully embedded within the device) to highly invasive (e.g. surgically implanting electrode arrays in the motor cortex) [15]. The richness of information is related to both the variety of discernible activities and the specificity of motion intention obtainable through a given modality.

The optimization to be performed is to maximize the richness of information while minimizing the invasiveness of the required instrumentation. From a practical standpoint, the error threshold for correctly identifying the user’s motion intentions needs to be such that he neither gets frustrated (or potentially injured) by incorrect estimates, nor feels like a Christmas tree due to the “decoration” of one’s self with a multitude of sensors with each donning and doffing of the device. The level of invasiveness required must also correspond to the severity of the morbidities stemming from the underlying condition. Societal acceptance and cosmesis are also critical practicality issues [44].

Here, a summary is provided exclusively for the sensor modalities that have been documented in the literature in the context of lower limb P/O control, organized by the level at which the user’s intentions are sensed.

#### Supraspinal neural activity

Recalling that motor intentions originate at the cortical level, several groups have investigated methods for triggering the device to provide assistance through Brain-Computer Interfaces (BCI) [45]. Recording of activity at this level has the potential to allow for a wide-variety of volitional movements, however, these may be difficult to decipher given that the brain is concurrently responsible for a multitude of tasks, including the control of the other limbs. In addition, many of the control loops responsible for physiological locomotion take place at the spinal level via reflex arcs (Figure 2), which may fundamentally preclude the use of neural activity to directly control the legs while maintaining balance during a dynamic task. However, there may still be utility in using brain activity to provide high-level commands to the device, which it will then execute (as in the *shared control* context promoted in [45–47] and demonstrated in [48, 49]).

Functional near-infrared spectroscopy (fNIRS) uses optical light emitters and receivers placed on the scalp to sense the haemodynamic response of the brain, which correlates with brain activity. This modality is subject to non-specific brain activity, motion artifacts, significant haemodynamic delay, and requires that optodes be worn on the head. Even so, a recent pilot study investigated the use of an fNIRS-BCI to detect the preparation for movement of the hip in seated stroke subjects, which may indicate its suitability in shared control with severely impaired subjects [50].

Electroencephalography (EEG) uses an array of surface electrodes to non-invasively record the electrical activity of the brain as evident on the scalp [45]. The EEG electrode arrays typically used in research are built into a snug-fitting skull cap that can be extremely difficult and time-consuming to put on by oneself, especially for the patient groups whose injuries would necessitate direct cortical input to the P/O controller. This supposedly could be countered with advancements in self-contained EEG headsets designed for consumer use. The electrodes can be either dry or wet, depending on whether an electrically conductive gel is required. Signals recorded via EEG can encode a wide variety of movements with high temporal resolution.

In practice, the use of EEG signals demands a high level of focus and concentration from the user and is susceptible to movement artifacts, autonomic neural activity and electrical noise. Use in real-world environments is further complicated by the presence of distractions and the performance of tasks that are unrelated to locomotion. However, EEG signals could be combined with other sensory inputs in the framework of the so-called hybrid BCIs [51, 52] in order to decode user’s high-level commands more reliably.

Environmental sensing (see section below) can add an additional layer of safety in the context of shared control with BCIs, as the controller may prevent certain movements due to the presence of obstacles [47]. For example, prior to executing a high-level command (e.g. go forward, turn left), the controller would check first whether there are any terrain features in the way. Similarly, the execution of the high-level command “sit down” would not require the user to align perfectly with the chair, but would rely on the controller’s ability to compensate for the misalignment. As these examples illustrate, shared control reduces cognitive workload, as the user does not need to care about the mid-to-low-level execution over long periods of time or during critical operations.

Implanted electrode arrays within the motor cortex enable measurements which may encode a wide variety of movements, with the noted downside of requiring a highly invasive (and still experimental) surgical procedure [15, 53, 54]. Such an interface may also be used to provide sensory feedback to the user, thus closing the sensory-motor control loop [54]. Intracortical electrode arrays have been successfully demonstrated to allow control of multi-degree-of-freedom reach and grasp movements with robotic arms in tetraplegic subjects [55, 56], though to date there are no known examples of cortically-implanted electrodes being used to control a lower limb device in humans. Similar experiments have been done, however, in rhesus macaques to demonstrate the prediction of leg movements to control of bipedal gait in a humanoid robot [57]. It remains to be demonstrated how well this technique would translate to the control of a wearable P/O device.

#### Peripheral neural activity

The closer that neural activity can be recorded to the innervated muscle, the more specific the motor commands become. Also interesting is the electromechanical delay between the motor commands and the generation of force in the muscle on the order of 10s of milliseconds [58], which would provide a significant head-start to a controller based on muscle activity over one based on mechanical feedback alone [59]. This delay, however, may also be a source of instability when a device with a faster control loop is coupled to the user to provide high levels of assistance [60].

These peripheral nerve signals can be sensed through the use of electromyography (EMG). Surface EMG is the least invasive technique, where electrodes are placed on the skin over the muscle belly of interest. Assuming that the musculature remains somewhat constant and that the device can be fastened to the body in a consistent manner, it may be possible to embed the electrodes within the human-robot physical interface, thus significantly reducing the amount of time required to don and doff the device [61, 62]. Surface EMG activity is susceptible to changes in electrode-skin conductivity, motion artifacts, misalignment of the electrodes, fatigue, and cross-talk between nearby muscles [60, 61, 63]. Myoelectric signals are also non-stationary in nature during a dynamic activity, which necessitates the use of pattern recognition techniques [64]. In practical use, a calibration routine is typically necessary each time the device is put on [60, 65].

In the event that a limb has been amputated, the residual neuromusculoskeletal stucture must be surgically stabilized. Depending on the location of the injury, the muscles responsible for the actuation of the amputated joints may still be present and natively innervated, albeit relocated and fixed to the bones in a non-physiological manner. In this case, it may be possible to record the EMG signals in the residual leg for the control of a particular joint (e.g. using muscles in the lower leg to control the ankle [66]). If the amputation is more proximally located (e.g. above the knee), the muscles to control the distal joint (e.g. the ankle) are altogether missing, and thus can not be used directly. However, given that the nerves that would normally control these muscles are still present, a technique called “targeted muscle reinnervation” (TMR) can be used [64, 67]. For TMR, the severed nerves are surgically reattached and allowed to reinnervate a foreign muscle, which can then be used as an EMG recording site for the amputated muscle. The reinnervated muscle acts as a “biological amplifier” for the severed nerve and provides a means to record its activity noninvasively via surface electrodes.

#### Joint torques and positions

Mechanomyography (MMG) can be used to estimate the force production in muscle by measuring the sound or vibrations evident on the surface of the skin using microphones or accelerometers [68]. A potential advantage of MMG over EMG is that the muscle force estimated through MMG is less sensitive to fatigue [69]. Force production can also be estimated via changes in muscle hardness [70, 71] and the volume of the muscle [72, 73]. A substantial downside to all of these approaches is their high sensitivity to motion artifacts, which may be significant given the nature of the physical coupling at the user-device interface.

Joint torques can be estimated via inverse dynamics provided measurements of the joint positions and external forces being applied to the limbs. Wearable sensors for estimating joint positions or limb segment orientations are summarized in [74] and include goniometers, inclinometers, accelerometers, gyroscopes, magnetometers, and inertial measurement units (IMUs). Ground reaction forces can be sensed using instrumented insoles worn under the foot (reviewed in [75]) or e.g. by measuring the load in the shank of a prosthesis. A variety of foot switches can also be used to deliver binary ground contact information, for example using force-sensitive resistors, sensed air pressure in a sealed tube under the foot, or a physical switch.

Furthermore, interaction forces can be measured at the physical interface between the user and the device. Useful sensors may include load cells, strain gages, pressure sensors, and force-sensitive resistors.

#### Alternative input modalities

Simple manual inputs (e.g. keypads, buttons or joysticks) may be effective even though the used signals are completely artificial [76, 77]. Voice commands or eye movements sequences have also been demonstrated as possible ways to interact with P/O devices [78–80]. Here again, the seamlessness and intuitiveness of these input methods are suboptimal, but they can represent viable alternatives when no input other methods are possible.

### Artificial sensory feedback and substitution

In the nominal sensory-motor system, sensory feedback from proprioceptors, exteroceptors, and the vestibular and visual systems close the physiological control loop, allowing stable and efficient locomotion, while also triggering supportive reflexes. Following neurological pathologies or amputation, this sensory feedback may be diminished or disrupted.

While it is possible to restore locomotive functionality without this information, artificial sensory feedback is necessary for the seamless integration of the P/O with the impaired sensory-motor system [81]. Feedback modalities may be either invasive or non-invasive, devices are stationary or portable, with the latter being more relevant for every-day use in combination with a P/O. A recent review has summarized the clinical impacts of wearable sensing and feedback technologies for normal and pathological gait [74], though the scope does not include their application to P/O devices.

Artificial feedback can be used for sensory substitution or augmentation. Sensory *substitution* replaces a lost sensor modality with another modality, e.g. by providing a sense of touch after amputation of the upper [82, 83] or lower [84] extremity. Sensory *augmentation* complements attenuated information using the same or a different sensor modality, e.g. visual feedback about the movement of a passively guided or prosthetic limb. Both sensory substitution and augmentation exploit brain plasticity, and different sensory modalities can be used to convey information and thereby restore function.

For non-invasive feedback, three major sensory channels are used: visual, auditory and tactile. Visual cues can convey diverse information, and can be projected, for example, on a screen or on the ground, or can be presented via virtual reality goggles. The visual channel already serves important functions during gait and other activities, which makes it susceptible to overloading. In addition, most of the visual feedback systems documented in studies are not portable, which may limit its feasibility to rehabilitation and training in controlled environments [85, 86] rather than everyday life. However, information about the center of pressure [87] or gait asymmetries [88] can be visualized on a portable device, for example using a smart phone or headset. In these studies, a significant modulation of the gait pattern was found when visual feedback was provided. Interestingly, subjects also indicated a preference for visual over auditory and vibro-tactile feedback.

Another commonly used sensory channel is hearing. Auditory cues can vary in stereo balance, pitch, timbre and volume [89], and therefore may transmit rich information via speakers or headphones. The auditory channel is also subject to overloading, and thus has limited suitability for everyday use. It may even be possible that relevant information, e.g. the sound of an approaching car, is masked. Even so, there are some studies that implemented and evaluated auditory feedback. In [88, 90, 91], for example, acoustic signals sounded when the gait symmetry ratio (i.e. ratio of time spent on right foot vs. left) exceeded preset thesholds. Differences between pre- and post-test symmetry ratio and a postural sway metric indicated that the subjects successfully incorporated the feedback to alter their gait. Gilbert et al. [92] acoustically displayed the knee angle of a prosthesis to above-knee amputees. Two of the study participants appreciated additional information; the third terminated the study as the employed feedback system drew unwanted attention from bystanders. This result is also telling of a social-acceptance hurdle that wearable P/O devices, including their sensors and feedback systems, must clear.

The tactile sense can be used to transmit low-dimensional information, and offers a variety of interfaces for feedback systems. Tactile cues can vary in frequency, strength, duration, pattern, and location [93]. The majority of feedback systems transmit discrete information [94–96] but moving stimuli are also possible [97, 98]. Electrotactile [94, 95, 99, 100] and vibrotactile [87, 101, 102] stimulation have been used to convey information about characteristics of gait and postural control and possible deviations. Sabolich et al., for example, successfully demonstrated in 24 lower-limb amputees that their “Sense-of-Feel” feedback system had positive effects on weight bearing and gait symmetry. Other tactile feedback schemes have been tested to display, for example, information about discrete force levels underneath the foot [103]. Perceptual testing with an unimpaired and an amputee subject was promising, however, the complete feedback system using balloon actuators has not yet been tested.

Besides non-invasive feedback systems, it is also possible to directly deliver electrotactile stimuli to peripheral nerves via implanted electrodes [83, 84]. For example, Clippinger et al. conveyed information about heel strike and bending moments in lower-limb prostheses [84]. Twelve patients were fitted with this system and qualitatively reported increased confidence during walking.

As stated previously, artificial feedback about the state and action of the assistive device should ideally not increase the cognitive load on the user. Therefore, it is important to determine the minimum information needed to improve the interaction with the device. This is nontrivial as it requires knowledge about the nominal role of sensory feedback in human postural and locomotion control.

Lower-limb prostheses have, for example, been equipped with embedded sensors to measure the pressure distribution underneath the prosthetic foot [95, 103], the location of the Center of Pressure (CoP) [87], the knee angle [94], or to detect gait events such as heel strike [91]. The choice of information to convey is mainly based on subjective experience and theoretical assessment of motor control.

Experimentally assessing [104, 105] or simulating [106] the user’s interaction with the orthotic or prosthetic device in conjunction with a feedback system may increase our understanding of which types of information are meaningful, superfluous or even incriminatory. Only intensive long-term testing and training in the real world will reveal whether artificial feedback truly closes the cooperative human-machine control loop, and thus allows for the efficient, safe and effective use of powered P/O devices.

## Environmental interaction

The environment provides the reaction forces responsible for the balance, support, and propulsion of the P/O user. These forces are a function of the ground contact surface condition, the slope, and the elevation of the terrain. Other forces arise due to the physical properties of the environment, such as gravity and fluid dynamic drag. *Obstacles* are terrain features that impede motion in a particular direction, thus forcing the user to circumnavigate or to perform a compensatory motion to negotiate. Each of these environmental properties have a great influence on the stability, balance, and energy consumption of the device and of the user [18] and thus should be considered in the overall control scheme.

The state of the environment can be indirectly inferred based on the states of the user and of the device or directly estimated using sensors explicitly for this purpose. This provides contextual information that can be used for the strategic implementation of control policies over a time window of several steps, as well as tactical information that can directly influence the control behavior within the current step.

### Implicit environmental sensing

It may be possible to discern certain environmental features from the states of the user and of the device at various instants of the gait cycle. Note the distinction between the identification of environmental features and the recognition of the activity mode: listed here are cases where the *properties* of the terrain are identified, which may subsequently be used e.g. for activity mode recognition.

When the heel and toe of the foot are in static contact with the ground, the slope can be estimated using an accelerometer mounted on the foot [107–109]. Given that there is no slip, the acceleration vector will match that of gravity, which can then be compared with the orientation of the sensor to give the slope. An IMU comprised of accelerometers and gyroscopes can be used to detect an elevation change of the ground between successive steps [109–111].

### Explicit environmental sensing

Scandaroli et al. presented a method using gyroscopes and infrared sensors [112] for estimation of the ground slope and elevation of the foot above the ground. In this application, two single-axis gyroscopes and four distance-measuring infrared sensors were mounted underneath a prosthetic foot. So far, only bench-top test results have been presented. Zhang et al. presented a “Terrain Recognition System” comprised of a body-worn laser distance sensor and IMUs fixed to the limbs [113]. The system estimates the height and slope of the terrain and was tested using an unassisted, able-bodied user with the laser sensor attached to the waist. An array of sonar sensors and digital video cameras was used to detect obtacles, which was used in the shared control context allow/disallow user commands with a brain-controlled wheelchair [47]. This approach could easily be extended to P/O devices.

Relatively few examples were found regarding active lower limb P/O devices that include explicit environmental sensing and adaptation, which is likely attributable to several factors. One is that many of the documented devices are still confined to well-defined and controlled environments as imposed by hardware and experimental constraints. Another is that much of the controller development has so far focused on the mastery of executing a particular task in a particular setting. Also possible is that sensors appropriate for environmental sensing have only recently become available and practical for use in a portable device. As each of these aspects attain sufficient technological maturity to provide generalized assistance that is responsive to real-world settings, it is expected that sensing of the environmental state and its physical and signal-level influence on the user, the device and the controller will gain higher priority.

### Environmental context

Knowledge regarding the setting through which the user moves is useful for strategic control planning because it constrains the likelihood of encountering a particular terrain feature and the degree to which the environment is structured. Within certain contexts, the environment can be regarded as quasi-static – that its properties remain somewhat constant over time until a new setting is entered. The exception to this would be an unstructured environment containing erratically located obstacles (e.g. a rocky hiking trail, a child’s messy room) or with variable surface conditions such as snow, sand, or loose gravel.

As an example of how contextual information could be used, when the user is inside a modern public building, the floor is typically flat and level, stairs are regularly spaced, and accessibility ramps will have a slope that is bounded by local construction codes. Thus, if a device is capable of localizing itself to within such a context, the decision space for high-level activity mode recognition can be weighted or reduced and the mid-level controller can be optimized for the most likely terrain. Such knowledge is also useful in a shared-control context, where the device is responsible for execution of the user’s high level commands.

There are currently no known examples where the environmental context has been used in P/O control. Nevertheless, such information could prove to be extremely valuable and is suggested as a future avenue of research.

## Control strategies

As depicted in Figure 1, the controller for the P/O device can be subdivided into three parts. The high-level controller is responsible for perceiving the user’s locomotive intent based on signals from the user, environment, and the device. This information is all passed to the mid-level controller, which translates the user’s motion intentions to a desired output state for the device. This command delegated to the low-level controller, which represents the device-specific control loop that executes the desired movement.

It is noteworthy that there are relatively few studies that document the implementation of a complete hierarchical, multifunctional control structure similar to the one suggested here and have demonstrated its use in a practical setting [24, 67, 114–119]. Instead, most studies focused on a particular subset one or two of these, typically the mid- and low-levels. It is contended that, for practical applications in the context of multimodal ADL, the majority of powered lower-limb P/O controllers will eventually adopt a structure that can be described by that of Figure 1.

### High-level control

The purpose of the high-level controller is to perceive the locomotive intent of the user through a combination of *activity mode detection* and *direct volitional control*. Depending on the user’s underlying pathology, the ability to generate, transmit, and execute appropriate locomotor commands may be impaired at some level. Therefore, once the user has provided a high-level command, the device should be responsible for the execution of movement via the mid- and low-level controllers. This *shared control* approach limits the cognitive burden imposed on the user [45, 46].

The desired high-level control output allows for the device to autonomously switch between different locomotive activities, ideally without imposing any conscious inputs from the user. Activity mode recognition can be coupled with direct volitional control to provide the user the ability to modulate the device’s behavior within a particular activity [120]. It is also possible to provide direct volitional control of the device in the absence of activity mode recognition.

#### Activity mode recognition

Activity mode recognition is what enables the high-level controller to switch between mid-level controllers that are appropriate for different locomotive tasks, such as level walking, stair ascent, and standing. The cyclic nature and long-term repeatability of various modes of gait lend themselves to automated pattern recognition techniques for classification. The inputs to the classifier include the sensed states of the user, the environment, and of the device. Important considerations for choosing a classifier include the number of activities from which to choose, the procedure required for training, its error rate in real-world conditions, signals that are required as an input, and the *classification latency* i.e. the time required by the classifier to reach a decision.

As useful definitions, Huang et al. coined the term *critical time* to describe the time by which a classification decision must be reached to ensure proper kinematic and kinetic transitioning between modes [59]. Thus, the classification latency must be shorter than the critical time to execute a proper transition. The critical time is an especially important constraint when transitioning between activity modes with substantially different characteristics, for example level walking to stair ascent, where excessive latency may cause a loss-of-balance. In subsequent work, Zhang et al. use the term *critical error* to describe any error that results in the subjective feeling of unstable balance [121]. This definition emphasizes not only that a loss-of-balance is to be avoided, but that the user must also feel secure with the performance of the device.

First, different types of classifiers that have been used for activity mode recognition will be discussed, then the sources of information that have been used as inputs to these classifiers will be presented. For additional information related to these topics, see the review of Novak and Riener [122] on sensor fusion methods in wearable robotics.

##### Heuristic rule-based classifiers

are a very simplistic, but fairly effective method for identifying mode transitions. Examples include finite state machines (FSM) [107, 114, 115, 117, 123], and decision trees [109, 113, 124–126]. Each of these methods operate using the same principle: given the set of all possible gait modes, the designer identifies a fixed set of rules that indicate the transition from one gait mode to another. These rules may be based on the sensed state of the user, device or the environment at a given point in the gait cycle. For example, a transition from level walking to stair ascent could be indicated by a sufficient change in elevation of the foot from the beginning of one step to the next [109]. In another, an iteration of the HAL-3 orthosis controller used a set of rules based on the sensed ground reaction force and the positions of the hip and knee joints to identify sitting, standing and walking [124].

Note that while the rules themselves in this case have been selected heuristically, the criteria used may either be manually selected [124] or determined through analytical means [109, 126]. Hysteretic thresholds can be used to prevent the device from inappropriately switching back and forth between modes, and must usually be set manually [107]. The latency of a rule-based classifier depends on how precisely the relative time within the gait cycle can be determined, thus up to a one-stride delay is typical, albeit potentially unacceptable, for certain transitions. The number of rules and thresholds that must be established increases nearly combinatorially with the number of gait modes (i.e. neglecting unlikely transitions, like stair ascent to sitting), and it is likely necessary to manually tune these parameters for a particular user [114]. Clearly, the heuristic rule-based approach is not scalable beyond a handful of very distinct activities and would be cumbersome to retrain as the user adapts to the device, potentially regaining locomotor capabilities over time.

##### Automated pattern recognition

techniques, rooted in the fields of machine learning and statistics, have yielded a variety of classifiers that can be used for activity mode recognition. Here, “automated” refers to the generation of classification decision boundaries during training (i.e. the classification itself is automatic even for the rule-based classifiers discussed above). Once supervised training has been completed on a representative data set, the classifier can be used to assign a class to a newly observed set of data based on its features. The decision boundaries may be linear or nonlinear, depending on the classifier. The inputs to the classifier may include the sensed state of the device, the environment and the user.

The clear benefit of using an automated classifier over one based on heuristic rules is that data from a multitude of sensors can be input to the classifier, from which additional features may be computed and used to make classification decisions that are less biased and potentially more accurate due to the high-dimensional input. Manual identification of these decision boundaries would likely be intractable otherwise.

The biggest shortcoming of this approach is the necessity of properly classified training data for all of the desired activities and the transitions between them, preferably incorporating sufficient variability such that the classifier will perform well in real-world scenarios. Furthermore, optimal classifier performance often requires training data from the user himself, which may be somewhere between difficult, impractical, and impossible to obtain [24, 127]. Training of the classifier can be greatly facilitated through the use of standardized tools and procedures, such as the “Control Algorithms for Prosthetic Systems (CAPS)” software used by the University of New Brunswick and the Rehabilitation Institute of Chicago [119, 128].

Examples of such classifiers that have been demonstrated with lower limb P/O devices include Naive Bayes [111], Linear Discriminant Analysis (LDA) [127, 129–131], Quadratic Discriminant Analysis (QDA) [132], Gaussian Mixture Models (GMM) [24, 49], Support Vector Machines (SVM) [59], Dynamic Bayesian Networks (DBN) [67, 133], and Artificial Neural Networks (ANN) [129, 134, 135]. Consideration of the relative merits and disadvantages of these classifiers and the mechanics of the classification process are beyond the scope of this paper.

All of these classifiers require *a priori* offline training, preferably conducted by the user himself. Young et al. explored the possibility of generalizing an activity mode classifier that is trained on one group of users and applying it to a novel user, with generally dissatisfying results regardless of the input source of the classifier [127]. However, the classification accuracy improved substantially when the classifier was “normalized” to the novel user by including some of his own level-walking data in the training set. Classifier accuracy can also be significantly improved when transitions are included in the training data in addition to steady-state data [119].

##### Inputs to the classifier

regardless of classifier type, can come from any number of sources, including the sensed states and interaction forces between the user, the environment, and the device. The required sensors may be built into the structure of the device itself, worn on the surface of the body, or implanted within the body, as discussed in a previous section. Here, the sources of information that have been used for activity mode recognition in portable powered assistive devices for lower limbs are considered.

Embedded mechanical sensing provides estimates of the device’s state to the classifier, and is an appealing approach because the required instrumentation can be fully integrated with the device itself i.e. does not have to be donned separately [24, 136]. Such signals include joint positions and torques, segment orientations and velocities, and ground reaction forces. For example, Varol et al. [24] employed a GMM to switch between sitting, walking, and standing modes using the embedded sensors in an actuated transfemoral prosthesis. LDA was used to reduce the dimensionality of the input feature set. The frame lengths were then optimized to yield high classification accuracy acceptable latency. The authors showed that following an initial 2-hour training procedure, the classifier remains accurate across several days of testing and despite sudden changes in the subject’s mass. Subsequent work has proposed the extension of this classifier to include standing on inclined surfaces [108], running [137], and stair ascent [138].

Environmental sensing was presented in an earlier section, and provides valuable information to the controller regarding the upcoming surface conditions, terrain, and context. This information has also been used to trigger an activity mode transition [107–110, 112, 113]. Environmental information provides an additional layer of safety in the context of shared control, where the controller is partially responsible for allowing/disallowing certain movements [47].

Body-worn force and position sensors, as discussed previously, provide estimates of the user’s state that can be input to a classifier. These can also provide useful information at times when the device’s state is ambiguous. In principle, some of these sensors could be embedded within the device [111]. For illustration, Novak et al. document a method for predicting the initiation and termination of level gait in real-time using 9 IMUs distributed about the body and pressure-sensing insoles via classification trees, with promising results [126]. So far only unimpaired and unassisted subjects have been tested, so it is unclear how well this would translate to assisted or pathological gait.

Movement of the Center of Pressure (CoP) or Center of Gravity (CoG), as projected onto a virtual ground plane, provides another means for the user to indicate their motion intentions. For this method, it is assumed that the user is capable of voluntarily shifting their body weight in both the frontal and sagittal planes, potentially through the use of a walker or forearm crutches. Following an appropriate shift in the CoP, a mid-level controller is called upon to execute the desired motion. This approach has been demonstrated in hip and knee orthoses for assistance following spinal cord injury for level walking [4, 117, 139–142] and for ambulation of stairs [143], and have thus far been implemented using heuristic rules-based classifiers. In most of these cases, movement of the CoP or CoG are also used as inputs to a mid-level finite-state controller, as will be discussed later on.

Sensing of cortical activity may be useful since physiological motion intention is ultimately rooted in the brain. Thus it makes sense to look at brain activity for high-level control. Shared control, which was originally described and successfully implemented with brain-controlled wheelchairs for severely impaired patients [45, 47], lends itself well to this purpose. EEG-based activity mode recognition has only recently been deployed with portable lower limb orthotic devices [48, 49, 144, 145].

Surface EMG provides a physiologically intuitive way to trigger activity mode transitions, even before an externally observable movement can be executed [129]. Au et al. demonstrated a neural network to switch between level walking and stair descent in an ankle prosthesis based on activation of the gastrocnemius and tibialis anterior muscles [134]. Tkach et al. used LDA to control a virtual 3-DoF ankle prosthesis using signals from multiple muscle groups in the upper and lower legs [146]. Jin et al. demonstrated the classification of six different activity modes based on features calculated from the myoelectric signal from three muscles [125]. Huang et al. implemented a phase-dependent LDA classifier to classify seven movement modes based on 16 channels of EMG input [129].

Neuromuscular-mechanical fusion was first documented in a subsequent study by Huang et al. [59] as a means to improve classification accuracy and speed beyond that which is possible using EMG [129] or mechanical signals alone [24]. The technique has been replicated by collaborators at the Rehabilitation Institute of Chicago (RIC) with a powered transfemoral prosthesis [127] and a powered transtibial prosthesis [131]. In later work at RIC [62, 67], a DBN classifier was used with the transfemoral prosthesis in place of the SVM or LDA of [59, 127]. The motivation for doing so is that a DBN (which is similar in concept to a hidden Markov model) uses prior sensor information that can be mixed with current information in order to estimate the likelihood of a transition between locomotive modes.

Note that with the EMG-based approaches listed above, the excitatory signals from the muscles are not directly used to manipulate the device as with the direct volitional controllers below, but strictly to switch between mid-level controllers for a given activity.

Manual mode switching is an effective alternative to convey user intent to a device. This can be implemented through selections made on a remote control [76, 142], pushing a button or squeezing a lever [140, 147], and the execution of a particular sequence of finger [148], eye [79] or limb movements made with the device [136]. While these methods produce a nearly unambiguous and definitive classification of the desired activity, they require conscious input from the user and disrupt the nominal physiological processes. Nevertheless, these may represent the only viable options depending on the severity of the underlying condition. It is noted that several of these examples that use manual mode switching are commercialized devices.

##### Important considerations

regarding activity mode recognition include the latency and error rates that are tolerable for each of the possible gait mode transitions. At best, an incorrect or late classification results in suboptimal assistance from the device; at worst it can result in a catastrophic loss of balance. A study by Zhang et al. on the effects of imposed locomotion mode errors with a powered transfemoral prosthesis concluded that the impact on the user’s balance depends highly on the gait phase where the error occurs and the change in the amount of mechanical work injected by the device as a result of the error [118].

For transitions between gait modes with substantially different characteristics (e.g. level walking to stair descent), errors in activity mode recognition tend to be much more critical and may present a safety hazard for the user. Thus, while seamless transitions represent the ideal controller, practical safety considerations favor robust and unambiguous mode switching. Presumably, this is why many commercial devices favor the manual mode switching described above.

Regardless of the type of classifier that is used in the high-level controller, there is always a mid-level controller running underneath. As a result, in many cases the penalty for misclassification or delayed classification of a given activity is not catastrophic due to the similarities between certain gait modes, such as level walking and ramp ascent [24, 62, 67, 109, 118]. While the selected mid-level controller may be suboptimal, the user may be able to adapt and accommodate the misclassification.

It would also be very practical provide some form of feedback to the user regarding the mode switching as reassurance that the device has correctly identified the next intended movement, for example through auditory or vibratory feedback [76, 142] or via the other modalities discussed in the section on artificial sensory feedback.

#### Direct volitional control

Volitional control grants the user the ability to voluntarily modulate the device’s state. Such functionality is especially important in scenarios where the locomotive activity is irregular or noncyclic (e.g. walking in a crowd or standing and shuffling), in situations where foot placement is critical (e.g. stair descent, walking on rough terrain), and during nonlocomotive activities (e.g. repositioning legs while sitting, bouncing a child on one’s knee). It is emphasized for consistency that, while the volitional intent is determined at the high level, the conversion to a desired device state occurs at the mid level.

Myoelectric signals are an intuitive approach to volitional control since they are already present during voluntary movement of the user’s own limbs. Sensing of peripheral neural activity for control does come with limitations, as were highlighted in the section on sensor modalities for human motion intentions. Surface EMG has been demonstrated for this purpose in transfemoral prostheses [61, 120, 130, 132, 149, 150], virtual above- and below-knee prostheses [151], a hip and knee orthosis [152], knee orthoses [60, 153, 154], a transtibial prosthesis [66] and an ankle-foot orthosis [155].

EMG-based control approaches differ in the way that the myoelectric signals recorded from the various muscle groups are mapped to the desired device state. The simplest approach is to directly modulate the actuator’s torque based on EMG activity [63, 152, 155]. A more complex approach uses a neuromusculoskeletal model to calculate net joint torques from the EMG signals of joint flexor and extensor muscles [60, 149, 154, 156]. One can also map processed EMG signals to a desired joint position, velocity, or acceleration by using a model of the coupled user-device system [153] or to the set-point angle or stiffness of an impedance control law [61, 66, 130, 132, 151].

It is also possible to use the EMG signals to contribute an additional flexor or extensor torque to the nominal torque output by a mid-level controller. This was demonstrated to allow stair ascent in a transfemoral amputee with a powered knee prosthesis [150]. This approach combines the inherent stability of the underlying controller (e.g. in the absence of any myoelectric input) while providing moderate levels of volitional control to the user.

As the user acclimates to and is able to predict the output behavior of the powered assistive device, it may be possible for him to volitionally manipulate the device by providing the appropriate set of inputs, possibly involving contrived or compensatory movements. This is likely true for mid-level controllers based on correlated postures [157] and invariant trajectories [158, 159] as discussed in the following section, though long-term studies would be required to show that users can learn to control the device in this manner.

### Mid-level control

The purpose of the mid-level controller (Figure 1) is to convert from the estimated locomotive intent output from the high-level controller (i.e. activity mode recognition coupled or direct volitional control) to a desired device state for the low-level controller to track. In many cases, there will be multiple mid-level control laws to accommodate the various activity modes. This controller may take as inputs the sensed state of the user, the environment, and the device.

An important differentiator between mid-level control implementations is the combination of temporal information, user or device states that are used to determine the gait phase. In some cases, the controllers do not even explicitly account for timing or the gait phase. Controllers which depend on the gait phase are referred to as *phase-based*, while controllers that do not depend on the gait phase are called *non-phase-based*. One implication of the phase-dependency is whether it is possible for a high-level controller to switch between activity modes within one gait cycle, or whether this can only occur at the beginning of the next cycle.

The input-output form of the control laws used in the mid-level controller have a profound impact on the device’s ability to interact with the user and the environment in a stable and purposeful manner. As such, the different forms of control laws will be discussed within this section.

The mid-level controller is also responsible for the coordination of control between multiple actuated joints, whether contained within one device or across multiple devices. It is also important to consider the contributions of the user toward locomotive dynamics. Coordinated control and load sharing are treated at the end of this section.

#### Phase-based controllers

In time-based control, a set of actions is performed based on a programmed time delay following a clearly identifiable gait event, for example heel strike [160, 161] or toe-off [162]. This technique is simple to realize and relies heavily on the regularity of the steady-state stepping period. As such, the weakness inherent to time-based control is its inflexibility to accommodate irregular or unprogrammed gait patterns (e.g. walking in a crowd or over rocky ground), unexpected events such as tripping, or within-cycle switching of activity modes [163].

Invariant trajectories represent user states which vary with respect to the gait phase, and do not change substantially with respect to gait speed, between different users, or with minor intra-gait-mode variations [164, 165]. By projecting a set of invariant trajectories onto judiciously chosen axes, one can derive an invertible relationship between the user’s state and the gait phase that is ideally independent of time, gait speed, or subject. While this technique does not represent a controller by itself, it can be used to input phase information to a mid-level controller without relying on the elapsed time between gait events, as with time-based methods. As such, walking backwards is also possible with this technique without altering the controller[158, 159]. It remains to be shown, however, whether these trajectories remain invariant when the user dons an actuated assistive device, across different modes of gait, or during pathological gait.

Normalized-trajectory control takes a prototypical joint trajectory from a set of previously recorded gait data and scales it to match the pace and physical size of the user as a function of gait phase. So-called “dynamic pace control” is one such example that uses the fast Fourier transform (FFT) to represent the prototypical trajectory by a set of Fourier coefficients. These can be used to scale and generate a desired device trajectory by taking the inverse transform [158]. One of the biggest challenges with this approach is identifying trajectories that can be appropriately scaled to both the speed and weight of the user. The output of the control law is typically a desired position.

Echo control is a combination of time-based and normalized-trajectory control, in which the position trajectories of the unassisted limb are recorded and replayed on the assisted limb with some time delay and scaling during steady-state reciprocal gait [114, 166, 167]. During certain activities (e.g. sit-to-stand transitions), no phase shift is required and the movement of the sound limb can be mapped directly to the impaired one. Such an approach assumes symmetry of movement between the two sides, and as such is inappropriate in cases where bilateral assistance is required or where the required stepping pattern is inherently asymmetric. Activities involving an odd number of steps can also be problematic. Note also that any undesired/compensatory movements that are recorded will also be replayed, which may result in instability or loss of balance. Gait mode switching can only be achieved at the beginning of a stride, and must be initiated with the unassisted limb.

Virtual constraint control is a strategy that has been used to control locomotion in bipedal robots [168], which has since been implemented and demonstrated with a powered knee and ankle prosthesis [159, 169, 170]. For this method, the anterior-posterior location of the CoP on the prosthetic foot is used as a phase variable, which is possible given that this trajectory monotonically increases throughout the stance phase of level walking. The so-called “effective shape” of the ankle-foot and knee-ankle-foot complex resembles a circular rocker when plotted against the CoP phase variable that is invariant with respect to speed, heel height, and body weight [165]. These effective shapes constitute virtual constraints that can be enforced through actuation of the device during stance, while a finite-state impedance controller (described below) was used during swing. The choice of a different phase variable may enable swing-phase virtual constraint control as well. As implemented, this technique enables walking patterns that are qualitatively similar to nominal ones using only generic, normalized shape parameters from literature (i.e. subject-specific tuning is not required).

**Figure 3:**
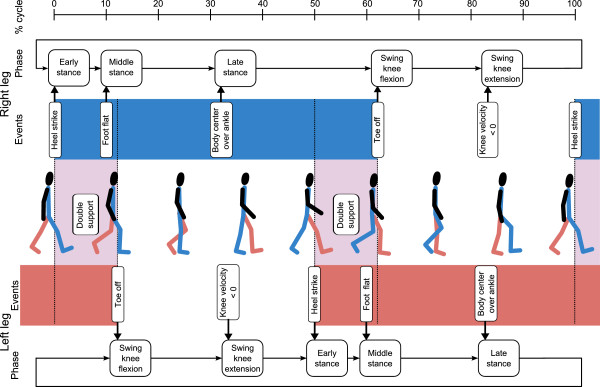
**Finite-state decompostition of level human gait.** Steady-state locomotion can be represented as a periodic sequence of states (or phases), where the transitions between the states are triggered by events within the gait cycle. The choice of the number of states and the type of events used are somewhat arbitrary, and will depend on what information is available from the sensors and which joint the P/O is to actuate. In this example for the knee joints, stance has been divided into three states, with early and middle stance initiated by ground contact events at the heel and toe of the foot, for example determined using pressure sensitive insoles. Late stance is triggered when the user’s center of mass is estimated to be over the ankle, again using the insoles or estimates of the user’s whole-body posture through joint position sensing or inertial measurements. Swing flexion begins as the toe of the foot leaves the ground, and swing extension begins as the knee’s velocity is sensed to be less than zero. The cycle begins again as the heel comes into contact with the ground.

Finite-state controllers (FSCs) decompose gait as a periodic activity that is described by a series of distinct phases, typically further delineated on the basis of foot contact events or joint velocities, as illustrated in Figure 3 and elaborated in the caption. A FSC implements a discrete set of parametric control laws that will cycle through as each new phase of gait is entered. These control laws differ in the way that the desired state of the device is computed, with popular choices being position and impedance control, as will be discussed below. A different FSC is required for each activity mode included in the high-level controller.

FSC is far-and-away the most popular mid-level control approach. Many groups have successfully implemented FSCs using different numbers of activities and states with a wide variety of devices [2, 3, 14, 24, 41, 44, 107–109, 114, 117, 118, 121, 123, 124, 134, 137–139, 143, 150, 163, 171–186].

The most of the referenced FSCs use a set of static parameters that are hard-coded into the controller, usually requiring a heuristic tuning routine involving the end-user. Given the inherent variability in the gait patterns between individuals, such tuning is likely necessary and indeed desired to optimize the user’s comfort and efficiency. However, this approach quickly becomes unwieldy, as the number of tunable variables rapidly increases with the number of parameters per control law, the number of states per activity, the number of activity modes, the number of joints to be actuated, and the number of limbs to be controlled [176].

To reduce parameter tuning time, Simon et al. documented the “modified intrinsic control strategies” to reduce the number of tunable parameters in a knee and ankle prosthesis by using impedance control laws based on joint position or load as opposed to a set of static parameters [187]. Aghasadeghi et al. demonstrated a model-based method to predict initial parameter values for a particular user based on invariant trajectories and his particular anthropomorphic characteristics [188]. Wang et al. describe the use of an expert system to automatically tune the impedance parameters to match that of healthy human gait based on fuzzy logic inference [189].

Furthermore, because these parameters are fixed in value, the P/O device may not optimally accommodate changes in the user’s gait, for example due to user fatigue, a sudden weight change, or variations within a particular gait mode. One approach to overcome this is to modulate a torque superimposed on the output of the control law through surface EMG [150]. Another approach implements a stance-phase control law based on a neuromuscular model, which was demonstrated to adapt to changes in both terrain [172] and gait speed [190] in a transtibial prosthesis.

#### Non-phase-based controllers

Complementary Limb Motion Estimation (CLME) infers the intended motion of affected limbs from the motion of the residual limbs, and maps this to a reference trajectory for robotic P/O joints to track [191]. This is possible due to the strong inter-joint coordination exhibited during physiological human motion [36]. The mapping is derived through regression of physiological gait recordings of healthy subjects.

CLME is sometimes confounded with echo control, as described above. By contrast, CLME complements residual body motion without delay. In addition, the recorded joints and their sensed states (e.g. position, velocity, acceleration) need not directly correspond to the controlled joint, as with echo control. For example, the motion of the arms and upper body are often highly correlated to the motion of the lower limbs, and thus represent potential recording sites for CLME-based control [192]. CLME does not depend on information of the gait phase, and no change of control is necessary between stance and swing. However, instrumentation of selected residual body parts must be provided.

CLME was first evaluated with the LOPES gait rehabilitation robot, showing that it can both replace (unilateral) leg function when needed [193], and be transparent when no assistance is needed [191]. Later, it was shown that an amputee subject can walk at different speeds and negotiate stairs [157] with a CLME-controlled actuated knee prosthesis. This study also demonstrated a qualitative reduction in the compensatory motion of the sound-side foot relative to a commercial controlled-damping prosthesis.

Recently, CLME has been extended to generate not only reference kinematics, but also stiffness. To obtain training data, active muscle stiffness during gait was quantified according to [194]. In a case study, an amputee subject successfully walked with CLME-controlled impedance [195].

In all of these studies, the training data used to generate the mapping came from a different subject than the controller was tested on. Even so, the controller was shown to be effective to restore functional gait patterns. This is an important consideration as it is often difficult to obtain nonpathological gait data from the user himself, which would contain the “ideal” joint trajectory mappings. CLME is only suitable for patients who can still control specific body parts, under the premise that these body parts are sufficiently correlated with the other limbs in physiological motion. Generalization of this method for different activities remains to be investigated.

Force-feedback control measures the interaction force between the user and the device and acts to reduce it according to an assistance ratio. Zero-force or transparency control, also given the colloquial name “get-out-of-the-way control”, is a special case of force-feedback control. It serves various practical purposes, especially with execution of non-locomotive tasks. The first purpose is for assisting the user with tasks that require force amplification while maintaining lower limb agility, as with exoskeletons for performance augmentation [11, 13, 196]. The second use would be to render the device to be transparent such that the user’s own movement is unrestricted. The renderable transparency of a device can also be used as a performance metric that is indicative of how well the physical dynamics of the device can be controlled [197–199].

#### Forms of control laws

The output from the mid-level controller is the desired state of the device. This state may consist of a combination of joint positions, velocities, and torques. The design of the device and the actuators it contains will have a strong influence on how well the desired state can be achieved, and thus must be taken into account during the development of the mid and low level controllers.

Commanding joint positions or velocities is a straightforward approach to robot control, for which an abundance of theory exists. In this case, the robot is tasked with the precise reproduction of a pre-defined trajectory. This type of controller works best when the output mechanical impedance (as defined below) of the actuator is high relative to the load, thus enabling the device to reject perturbations from the user or the environment. Controlling the position or velocity of the device is useful when the desired trajectory and the interaction forces are well-characterized; but both of these may be difficult to predict given the dynamic nature of locomotion. Interaction with stiff objects (e.g. the environment) can lead to instability and the generation of high forces under this type of control [200]. Presumably, it is because of these issues that very few examples of assistive devices for lower limbs were found that use position control.

The causal dual of angular velocity is torque. Torque control is possible when the output mechanical impedance of the actuator is low relative to the load, and is useful for providing assistive forces when the desired position is ill-defined or unimportant. Problems can arise, however, when torques are applied without regard to the position of the joint (e.g. uncontrolled motions, bumping into RoM limits).

Impedance is defined as the transfer function between an input flow and an output effort [201]. In the domain of mechanical rotation, controlling the impedance of a joint is to control the *relationship* between the accepted angular velocity and the yielded torque. Since the output of an impedance controlled joint is ultimately a torque, the output mechanical impedance of the actuator must be relatively low. A wide variety of virtual dynamics can be rendered under impedance control, with common intuitive examples being linear stiffness, damping and inertia. By applying passivity constraints to the rendered dynamics, the stability of the impedance-controlled system can be guaranteed [200, 202].

A leading theory regarding physiological movement is that the CNS controls the limbs through impedance control [203, 204]. It is partly due to property that human movements can be robust to perturbations despite the delays inherent to transmission of efferent and afferent signals via neural pathways, as explained in the section on nominal control of human locomotion. Thus, it is possible to realize a bio-inspired approach to P/O control through impedance modulation. An improved understanding of how humans control the impedance of the lower limbs is necessary to optimize the mechanical design and control of P/O devices and is the topic of ongoing biomechanics and neuroscience research [156, 194, 205–209].

Admittance is defined as the inverse of impedance, and thus also defines a relationship between effort and flow. Admittance control has been proposed as an approach to masking the undesired dynamics (e.g. added friction and inertia) imposed by an exoskeletal device [210]. This type of control has been used with treadmill-based gait orthoses [211], but no examples of admittance control implementations with a portable device were found.

It is impossible to say whether it is best to control a device’s position, velocity, torque, impedance, or admittance, though the choice may be constrained by the physical dynamics of the device and how it is actuated. There are distinct advantages and disadvantages to each type of control, and there may be different conditions and gait phases that lend themselves to switching between control modes [134, 185, 212].

#### Coordinated motion control and load sharing

Prosthetic and orthotic devices are part of an overall system involving physical interactions, and thus cannot operate as if in isolation. A coordinated mid-level control scheme is necessary whenever there are multiple actuated degrees of freedom, whether contained within one device [117, 170, 181, 213, 214] or distributed across multiple devices [176]. Uncoordinated motion between limbs and joints can result in loss of balance or falls, and so the states of each should be communicated and taken into account to prevent such an occurrence. Monitoring of the state of the user (e.g. ground contact state, joint positions) also provides relevant information to the controller.

It should also be noted that the joints in the human body are subject to biarticular coupling on both a mechanical and neural level [215]. As a result, the realizable voluntary torque output and range-of-motion (RoM) of the user’s joints are functions of posture, which potentially involves joints that are not specifically actuated by the device. Considering this, the mid-level controller should take into account the configuration of the user to ensure that the device does not over-power the joint nor exceed his RoM.

Recalling that an orthotic device typically acts in parallel to the user’s limb, it is possible (and indeed desirable, as per the ”Slacking Hypothesis" [20]) to make the user responsible for sharing a portion of the load carried at the joint. The fraction of the load borne by the device is termed the *support ratio* [60] or the *assistance ratio* [216], and is normalized to either the net torque required of the joint for a particular movement or to the maximum torque of the device. The load carried by the physiological joint can be estimated from muscle activity [60, 70, 154, 216] or through direct force measurement [217].

Care must also be taken that the device does not provoke unphysiological muscle activity, for example excessive cocontraction or off-nominal timing of muscle activation. Note that the net joint torque may look “normal”, even though the underlying muscular activity is not natural. This is an especially important consideration in the context of retraining gait patterns following neurological injury, and may be monitored e.g. via EMG.

In the wake of certain impairments (e.g. spinal cord injury), however, the transmission of voluntary locomotor commands is interrupted. Short of physiological recovery or bridging the communication gap with neuroprostheses [21], functional electrical stimulation (FES) can be used to actuate the user’s muscles in cooperation with an orthotic device [218, 219]. Such stimulation must be provided in concert with the user’s intentions and locomotive state and provides a means for the user to actively participate in the locomotive task with the support of the orthosis. One obstacle to every-day use of FES is the need to precisely place electrodes over the muscles, which may be difficult for the user to achieve without assistance. A mid-level controller, such as a FSC, must underly the FES to manage the movement of the device itself.

### Low-level control

The purpose of the low-level controller (Figure 1) is to calculate the error between the device’s current and desired states (i.e. the output from the mid-level controller) and to drive the actuator to reduce this error. This execution-level of control tends to be highly device-specific, and may rely on a combination of feedforward and feedback loops.

Feedforward control requires some form of model to predict the system’s future state based on the past and current set of inputs and device state. Such control inputs can be effective at reducing undesired interaction forces due to the added mass, inertia, and friction of the device [178].

Feedback controllers do not require a model of the system *per se*, but do require an estimate of the current state. The controller compares this with the desired state of the device and modulates the power input to the device to drive any discrepancy to zero. A wide variety of control techniques can be used to achieve this, the details of which are left to any reputable controls textbook. Many classical control strategies employ negative feedback, though the Berkeley Lower Extremity Exoskeleton (BLEEX) employs a positive feedback loop [220, 221]. In this example, the net effect of the positive feedback is to increase the controller’s sensitivity to the user’s interactions without requiring force sensing between the user and the device. The trade-off is that very precise models of the device’s dynamics are required for each of the bilateral ground contact states. This was counteracted using the hybrid control scheme outlined in subsequent work [214].

The physical configuration and dynamics (i.e. friction, inertia, actuator power output) of the device will fundamentally limit its ability to stably track a desired output trajectory. The performance of digitally-controlled systems is further constrained by sensor noise, signal quantization, discrete sampling effects, and control loop execution times. The use of passivity constraints (i.e. that the device is controlled to store or dissipate energy, but not add) provides a means to guarantee the stability of the device as it interacts with the user and his environment [182, 200, 202].

## The P/O device

The device represents the hardware embodiment of the robotic P/O. This includes the device’s physical structure, actuators, embedded sensors, control system, energy storage, and power amplifiers. While the mechanical design and implementation of P/O devices is beyond the scope of this review, the hardware must be taken into account as it heavily influences the low-level control possibilities. For example, it may be difficult and inefficient to render forces with an assistive device whose actuator output impedance is high [197], conversely the maximum torque output, controllable bandwidth and the dynamic range of renderable impedance (i.e. Z-width) are reduced for an actuator with low output impedance [202].

Additional considerations are the performance limitations and saturation effects of the actuator and power source [14, 60, 170]. Even state-of-the-art portable devices must be driven beyond their continuous operating range to achieve the power outputs required during energetically demanding activities, such as sit-to-stand, stair ascent, running, or jumping [61, 217]. While this is generally acceptable for short bursts, it may be necessary for the controller to derate the actuator if these conditions are sustained for long periods.

A further optimization for the controller may be the energy efficiency of operation. In addition to minimizing the energetic expenditure of the user, the device may also be tasked with minimizing its own energy consumption, thus extending its useful range of operation. Portable devices are inherently limited in their energy storage capabilities, and as such the conversion of power to a usable form should be as efficient as possible. Aside from improvements in amplifier or transmission technology, energy-saving approaches include the use of passive compliance, e.g. [3, 205, 222], mechanically-variable impedance actuation [223, 224] and the reciprocal transfer of power between joints [213, 225–228] or between portions of the gait cycle [229–231].

It is reiterated that for an active P/O device to be practical for daily use, both the mechanical design and the controller must be of sufficient maturity. In order to develop controllers that are optimized for unencumbered human-robot interaction, it would be advantageous to use devices whose energetic performance exceed that of the human body. Toward this end, Steven Collins [232] has called for the development of “universal wearable robot emulators” – devices for use in a laboratory that are tethered for power, control, and actuation (see e.g. [155, 162, 166, 206, 212, 233]). By removing the mechanical constraints, research can focus on the development of optimal controllers for use pending improvements in the power output capabilities of portable devices.

However, depending on where and how the opposite end of the tethers are mounted, a different set of constraints may be imposed on the types of movements that can be executed (e.g. turning direction and magnitude) or on the realism of the terrain that can be tested. To overcome these, it may be possible to develop a portable tethered emulator that either follows the user around (e.g. rolled on a cart [72] or carried by a second person) or is carried by the user himself in a way that may be impractical as a product, yet informative for research purposes.

Controllers can also be developed via numerical simulations [234] or robotic analogs [235]. These approaches come with the benefit that many different algorithms can be tested in a highly repeatable setting without ever risking a human subject. While extremely informative, the shortcoming is that these methods rely on models of the human control system, which is still heavily debated. These also may neglect certain “human factors” and the variability inherent to physiological locomotion.

## Safety mechanisms

Considerations of safety must underly nearly all aspects of the mechanical design and control of P/O devices. These must include safety of the subject, his environment, and of the device itself. This is especially important for powered devices, which may be capable of generating destructive forces and whose controlled output behavior may not always be in agreement with the user’s intent. The identification, quantification and mitigation of risks is thus a critical aspect of device development.

Common risks to locomotion include slipping, tripping and falling, all of which may be exacerbated by the presence of the P/O device. The cause of such incidents may be internal to the device (e.g. controller failure) or due to external factors, such as encountering unexpected terrain. The severity of these risks spans a wide range, and must be weighed against the probability that they will occur, and whether such a failure is detectable. The risks are also not limited to physical harm, but may also include social, emotional, and psychological effects [38, 39].

In early 2014, the International Standards Organization (ISO) published standard ISO 13482, which provides definitions, design guidelines, and safety requirements for personal care robots [236]. Though this standard expressly does not cover robots as medical devices (as many P/Os would be considered), it does apply to wearable suits and exoskeletons for physical assistance. Pending the development of safety standards specific to personal care robots for medical use, ISO 13482 perhaps provides an initial set of guidelines for the identification and assessment of risks in wearable robotic devices.

Some additional tools for risk identification and quantification include Failure Modes and Effects Analysis (FMEA) [237], Hazard and Operability (HAZOP) and Hazard Analysis (HAZAN) studies [238], and Fault Tree Analysis (FTA) [237]. All of these methods are semi-empirical and rely on the developers’ expertise in identifying potential causes of injury or damage, and thus may not capture all hazards that may arise.

Despite the paramount importance of safety in P/O design and control, the results of safety evaluations are rarely, if ever, reported in the literature. Thorough consideration of this topic is critical to the future of robotic P/O development, and thus should be given much more attention than is possible here.

Mitigation of risk involves the inclusion of passive and active safety mechanisms. Passive safety mechanisms are those which fundamentally limit the power transmission of the device without requiring any input power or feedback control. These include mechanical stops that limit the RoM, intrinsic force/torque limitations [206, 212], and electrical circuits with appropriate grounding and fuses. Switches to manually disable the device are themselves passive, though they require intervention from the user [212].

Active safety mechanisms are those that limit power transmission through feedback control, which typically requires input power. This includes configuration-dependent actuation torque and range-of-motion limitation as an active safety mechanism, as suggested in the discussion on coordinated control. Redundant sensing allows for the system to monitor it’s own health and to identify controller, actuator, or sensor failures. Upon detection of a failure, the controller implements a “safe behavior” (e.g. making the joints stiff or compliant), where the appropriate behavior must be decided upon by the controls designer [60, 114, 116]. These failures can be either momentary or persistent. In either case, the device should alert the user to the situation and provide means to recover or to reset the controller of the device [76, 136].

Examples of failures discussed in the literature include inappropriate controller outputs due to misinterpretation of user intent or invalid sensor inputs [24, 67, 116, 118, 121, 136, 176, 214, 239], unstable interactions with the user and environment [60, 107, 138, 181, 184, 202], actuation failures due to overloading/saturating the actuator [136, 170, 214], failure of an underactuated system to achieve its desired state, power failures due to a loss of power to the actuator or to the controller [136, 231], and controller failures due to software bugs or overloaded computational resources [60]. As a quasi-failsafe measure against falling, the commercialized ReWalk™ orthosis includes a wearable airbag system that can either be deployed manually by the user or autonomously using built-in sensors [142].

The user, as an integral element of the control system, is also partially responsible for ensuring safety. This includes the rational avoidance of potentially unsafe situations, but also the response to unexpected locomotive events, such as tripping, stumbling and slipping [114–116, 118, 175, 199, 240]. Falls represent a substantial hazard to the user, and so the device must (at minimum) not increase the risk of falling. Until advanced balance recovery controls have been developed, the appropriate response of the device in these cases is most likely to become transparent for the user to recover balance on his own [199].

For the commercialization and adoption of powered P/O devices in ADL, developers must establish trust with regulatory bodies, funding agencies, insurance companies, clinicians and end users that such devices are not only effective at their intended application, but are safe to use within reasonably expectable circumstances (see e.g. [241, 242]). This will typically necessitate clinical trials.

How, exactly, to establish the efficacy (e.g. in terms of ADL performance or rehabilitative outcomes) and safety of a device is beyond the scope of this paper. Based on the reviewed literature, however, we can echo some recent observations by Farris et al. [4] that there appears to be little standardization in the methodologies and outcome measures that are used to evaluate the efficacy of active P/O devices, and very few papers provide balanced criticism of the limitations own methodologies. Toward this end, an increase in clinical trials is required of developers, along with the creation of standardized evaluation criteria.

As a recent example, the ReWalk™ became the first FDA-approved powered orthosis to be marketed for personal use via the “de novo” classification process. This classification is reserved for novel devices for which there have been clinical studies and extensive performance testing, but clear effectiveness and safety evaluation standards have yet to be established. The use of this type of classification indicates that there is currently a regulatory gap that has yet to be filled.

Some parallels can be drawn between the fields of wearable robotics and surgical robotics in that these (potentially powerful) devices are working in intimate proximity to humans. As such, one may draw inspiration from the design framework for surgical robots recently presented by Sánchez et al., where an in-depth discussion of design methodologies, passive and active safety guidelines, and relevant certification standards is provided [243]. While the hazards associated with robotic surgery will typically garner higher risk classification than powered P/Os, many of the hardware and software design principles are universal.

## Conclusions

Through this literature review, the state-of-the-art in control strategies for portable, powered lower limb P/O devices has been established. The control strategies that have been used vary substantially in accordance with the intended application and functionality of the P/O device, the structure and scope of the control scheme that is implemented, and with the instrumentation necessary for sensing the state of the human-robot system. There may also be marked differences in the flexibility of the control algorithms to adapt to changing conditions with respect to the user and to the environment, and the ability to recognize and to make transitions between different locomotive activities. Though these differences may fundamentally preclude the direct translation of certain control paradigms between devices, there are also many concepts that can be applied universally.

These control strategies were presented in the framework of a novel generalized control framework. This accounted for the physical and signal-level interaction between all of the components of the active P/O ecosystem, including the user, the environment, the controller, and the device itself. This framework is suggested for future use in the holistic design of controllers for such devices.

The user was discussed with respect to his role as the pilot of such devices and as an integral part of the control plant. This includes consideration of the physiological systems responsible for control of nominal locomotion and the compensatory mechanisms exhibited during unassisted or assisted gait following neurological, muscular or skeletal injury. Various sensor modalities were highlighted for tapping into the user’s physiological control system. The chosen modalities must be appropriate for the user’s physiological condition and personal preferences.

To date, there have been relatively few long-term studies regarding the use of artificial sensory feedback in conjunction with powered P/Os and whether it may enhance the user’s sense of control and embodiment of the device. It is contended that the provision of such feedback is a necessary future step to achieving seamless integration of the P/O controller with the user’s sensory-motor control scheme.

The environment plays an important role in human locomotion as it places constraints on the movement possibilities. Sensing of the surface conditions, terrain, obstacles, and environmental context can provide valuable information to the P/O controller. So far, there have been few studies that specifically account for environmental conditions and context within their control architecture, but this is expected to change as more of these devices begin to operate in unstructured and real-world environments.

The generalized controller is represented as a three-tier hierarchical architecture that very loosely resembles the structure and functionality of the CNS, and is an effective way to decompose the task of controlled locomotion. As research in this field matures and shifts toward delivering multifunctional devices for daily use, this type of architecture is the most likely to be adopted. Shared control can be implemented within this structure, which delegates the cognitive burden of mid-to-low-level decision-making to the device.

The high-level controller is responsible for perceiving the user’s locomotive intent, which consists of activity mode recognition or direct volitional control. Determination of the user’s steady-state activity, as well as the transitions between them, requires the use of a trained classifier. Proper intent recognition is necessary for the controller to execute a response that is both appropriate for the task and corresponds to the user’s expectations. Advancements in machine learning techniques and the recent proliferation of wearable sensor technologies is likely to fuel developments in this area.

Direct volitional control allows the user to voluntarily modulate the device’s output behavior, and is of particular importance during non-periodic or non-locomotive activities and those which require precise positioning of the limbs. The combination of activity mode recognition with direct volitional control combines the robustness of steady-state activity detection with the ability to voluntarily modulate the limb movements for fine placement. This strategy is seen as the most promising approach to smooth and accurate multifunctional human-robot interaction.

The mid-level controller translates the user’s locomotive intent to a desired trajectory for the device to track. Depending on the type, these controllers may or may not depend on the user’s gait phase. FSCs are the overwhelming mid-level controller of choice, owing largely to their conceptual tractability and ease of implementation. They are not, however, without their shortcomings, particularly the exploding dimensionality of the tunable parameter space. The use of so-called “modified intrinsic control strategies” is a promising approach to minimize the number of tunable parameters.

The choice of input-output variables, along with the form of the mid-level control laws (e.g. admittance, impedance, or other), will determine the overall system behavior. Each of these forms is a valid choice for different scenarios. The coordinated motion between joints, whether human or robotic, is also the task of the mid-level controller and is critical to the safety and stability of assisted gait. Coordinated sharing of the load between the human and an orthosis may also be necessary to realize rehabilitative outcomes.

The role of the low-level controller is to realize the desired trajectory of the selected output variables as specified by the mid-level controller. This is typically achieved through closed-loop control, which may involve feedback or feedforward loops. It is at this level of control that the device’s kinematics and dynamics are taken into account and used to compute the set of actuator inputs to achieve the desired states in a dynamic, yet stable manner.

The hardware-realization of the device was considered in terms of its implications for control design, in particular the constraints that it places on real-world performance. Many of the existing portable powered P/O devices must be operated near the edge of their envelope of continuous operation during normal use. Advances in actuator, energy storage and power conversion technology, and efficient control strategies may eventually overcome many of these issues. Meanwhile, the use of remotely actuated devices will accelerate the development of unencumbered controllers until sufficiently powerful devices are available.

Safety considerations lie at the heart of all aspects of wearable robotic technology. Designers should view hardware and software failures as inevitabilities and should include fail-safe measures and redundant systems to prevent injuries to life or property. These considerations will be of critical importance to the development, social acceptance and regulatory approval of devices that are effective and appropriate for assisting persons with disability in locomotive ADL.

In summary, this field of research is entering an age where the technological maturity of both the hardware and software are sufficient to realize bionic lower limb P/Os that are practical and safe for real-world use. This would represent a substantial achievement in human history, and holds the potential to dramatically improve the quality of life for those living with impaired mobility. This can only be attained through continued collaboration and open communication between research groups, interdisciplinary cooperation between engineers, physiologists, clinicians, industrial partners, and end-users, and the expansion of funding opportunities from public and private entities.

## References

[1] Seymour R (2002). Prosthetics and orthotics: lower limb And spinal.

[2] Au SK, Weber J, Herr H (2009). Powered ankle–foot prosthesis improves walking metabolic economy. Robot IEEE Trans.

[3] Martinez-Villalpando EC, Mooney L, Elliott G, Herr H (2011). Antagonistic active knee prosthesis. a metabolic cost of walking comparison with a variable-damping prosthetic knee. Engineering in medicine and biology society (EMBS), 2011 Annual international conference of the IEEE,.

[4] Farris RJ, Quintero HA, Murray SA, Ha KH, Hartigan C, Goldfarb M (2014). A preliminary assessment of legged mobility provided by a lower limb exoskeleton for persons with paraplegia. Neural Syst Rehabil Eng IEEE Trans.

[5] Cohen JE (2003). Human population: the next half century. Science.

[6] The European Registers of Stroke (EROS) Investigators (2009). Incidence of stroke in europe at the beginning of the 21st century. Stroke.

[7] National Spinal Cord Injury Statistical Center (United States) (2012). 2012 Annual Report - Complete Public Version. Annual Report - Available Online.

[8] Van Den Eeden SK, Tanner CM, Bernstein AL, Fross RD, Leimpeter A, Bloch DA, (2003). Incidence of Parkinson’s disease: variation by age, gender, and race/ethnicity. Am J Epidemiol.

[9] Ziegler-Graham K, MacKenzie EJ, Ephraim PL, Travison TG, Brookmeyer R (2008). Estimating the prevalence of limb loss in the United States: 2005 to 2050. Arch Phys Med Rehabil.

[10] Herr H (2009). Exoskeletons and orthoses: classification, design challenges and future directions. J Neuroeng Rehabil.

[11] Guizzo E, Goldstein H (2005). The rise of the body bots. Spectrum IEEE.

[12] Dollar AM, Herr H (2008). Lower extremity exoskeletons and active orthoses: challenges and state-of-the-art. Robot IEEE Trans.

[13] Bogue R (2009). Exoskeletons and robotic prosthetics: a review of recent developments. Ind Robot: Int J.

[14] Shorter KA, Xia J, Hsiao-Wecksler ET, Durfee WK, Kogler GF (2013). Technologies for powered ankle-foot orthotic systems: Possibilities and challenges. Mechatronics IEEE/ASME Trans.

[15] Goldfarb M, Lawson BE, Shultz AH (2013). Realizing the promise of robotic leg prostheses. Sci Transl Med.

[16] Pons JL (2008). Wearable Robots: Biomechatronic Exoskeletons.

[17] Jimenez-Fabian R, Verlinden O (2012). Review of control algorithms for robotic ankle systems in lower-limb orthoses, prostheses, and exoskeletons. Med Eng Phys.

[18] Riener R, Rabuffetti M, Frigo C (2002). Stair ascent and descent at different inclinations. Gait Posture.

[19] Veneman JF, Kruidhof R, Hekman EEG, Ekkelenkamp R, Van Asseldonk EHF, van der Kooij H (2007). Design and evaluation of the lopes exoskeleton robot for interactive gait rehabilitation. Neural Syst Rehabil Eng IEEE Trans.

[20] Marchal-Crespo L, Reinkensmeyer D (2009). Review of control strategies for robotic movement training after neurologic injury. J Neuroeng Rehabil.

[21] Borton D, Micera S, Courtine G, Millán JdR (2013). Personalized neuroprosthetics. Sci Transl Med.

[22] Peckham PH, Kilgore KL (2013). Challenges and opportunities in restoring function after paralysis. Biomed Eng IEEE Trans.

[23] Triolo RJ, Bailey SN, Miller ME, Rohde LM, Anderson JS, Jr JAD, (2012). Longitudinal performance of a surgically implanted neuroprosthesis for lower-extremity exercise, standing, and transfers after spinal cord injury. Arch Phys Med Rehabil.

[24] Varol HA, Sup F, Goldfarb M (2010). Multiclass real-time intent recognition of a powered lower limb prosthesis. Biomed Eng IEEE Trans.

[25] Loeb GE (1989). Neural control of locomotion. BioScience.

[26] Stein PSG, Stuart DG, Grillner S, Selverston AI (1999). Neurons, networks, and motor behavior.

[27] Van de Crommert HW, Mulder T, Duysens J (1998). Neural control of locomotion: sensory control of the central pattern generator and its relation to treadmill training. Gait Posture.

[28] Duysens J, Van de Crommert (1998). Neural control of locomotion; Part 1: The central pattern generator from cats to humans. Gait Posture.

[29] Zehr EP (2005). Neural control of rhythmic human movement: the common core hypothesis. Exerc Sport Sci Rev.

[30] Dimitrijevic MR, Persy I, Forstner C, Kern H, Dimitrijevic MM (2005). Motor control in the human spinal cord. Artif Organs.

[31] Pons JL, Moreno JC, Torricelli D, Taylor JS (2013). Principles of human locomotion: a review. Engineering in medicine and biology society (EMBC), 2013 35th annual international conference of the IEEE.

[32] Dietz V (2002). Proprioception and locomotor disorders. Nat Rev Neurosci.

[33] Prochazka A, Gritsenko V, Yakovenko S, Stuart DG, Gandevia SC, Proske U, Stuart DG (2002). Sensory control of locomotion: Reflexes versus higher-level control. Sensorimotor control of movement and posture. Advances in experimental medicine and biology, vol. 508.

[34] Capaday C (2002). The special nature of human walking and its neural control. Trends Neurosci.

[35] Nielsen JB, Sinkjær T (2002). Afferent feedback in the control of human gait. J Electromyogr Kinesiol.

[36] St-Onge N, Feldman AG (2003). Interjoint coordination in lower limbs during different movements in humans. Exp Brain Res.

[37] Waters RL, Mulroy S (1999). The energy expenditure of normal and pathologic gait. Gait Posture.

[38] Miller WC, Speechley M, Deathe B (2001). The prevalence and risk factors of falling and fear of falling among lower extremity amputees. Arch Phys Med Rehabil.

[39] Rubenstein LZ (2006). Falls in older people: epidemiology, risk factors and strategies for prevention. Age Ageing.

[40] Gailey R, Allen K, Castles J, Kucharik J, Roeder M (2008). Review of secondary physical conditions associated with lower-limb amputation and long-term prosthesis use. J Rehabil Res Dev.

[41] Herr H, Wilkenfeld A (2003). User-adaptive control of a magnetorheological prosthetic knee. Ind Robot: Int J.

[42] Belda-Lois J-M, Mena-del Horno S, Bermejo-Bosch I, Moreno J, Pons J, Farina D, (2011). Rehabilitation of gait after stroke: a review towards a top-down approach. J Neuroeng Rehabil.

[43] Einarsdottir H (2011). Intelligent motor powered prosthetic knee joint. J Med Devices.

[44] Kobetic R, To C, Schnellenberger J, Audu M, Bulea T, Gaudio R, (2009). Development of hybrid orthosis for standing, walking, and stair climbing after spinal cord injury. J Rehabil Res Dev.

[45] Rupp R, Mueller-Putz G, Murray-Smith R, Giugliemma C, Tangermann M,, Millán JdR (2010). Combining brain-computer interfaces and assistive technologies: State-of-the-art and challenges. Frontiers Neurosci.

[46] Renkens F, Mourino J, Gerstner W, Millán JdR (2004). Noninvasive brain-actuated control of a mobile robot by human eeg. Biomed Eng IEEE Trans.

[47] Carlson T, del R Millán J (2013). Brain-controlled wheelchairs: a robotic architecture. Robot Automation Mag IEEE.

[48] Contreras-Vidal JL, Grossman RG (2013). NeuroRex: a clinical neural interface roadmap for EEG-based brain machine interfaces to a lower body robotic exoskeleton. Engineering in medicine and biology society (EMBC), 2013 35th annual international conference of the IEEE.

[49] Kilicarslan A, Prasad S, Grossman RG, Contreras-Vidal JL (2013). High accuracy decoding of user intentions using EEG to control a lower-body exoskeleton. Engineering in medicine and biology society (EMBC), 2013 35th annual international conference of the IEEE.

[50] Rea M, Rana M, Lugato N, Terekhin P, Gizzi L, Brötz D, (2014). Lower limb movement preparation in chronic stroke: a pilot study toward an fNIRS-BCI for gait rehabilitation. Neurorehabil Neural Repair.

[51] Pfurtscheller G, Allison BZ, Bauernfeind G, Brunner C, Solis Escalante T, Scherer R, (2010). The hybrid BCI. Frontiers in neuroscience.

[52] Mueller-Putz GR, Breitwieser C, Cincotti F, Leeb R, Schreuder M, Leotta F,: **Tools for brain-computer interaction: a general concept for a hybrid bci (hbci).** *Frontiers Neuroinformatics* 2011;.,**5**(30)**:** doi:10.3389/fninf.2011.00030 10.3389/fninf.2011.00030 PMC3223392 22131973

[53] Nicolelis MAL (2012). Mind in motion. Sci Am.

[54] Bensmaia SJ, Miller LE (2014). Restoring sensorimotor function through intracortical interfaces: progress and looming challenges. Nat Rev Neurosci.

[55] Collinger JL, Wodlinger B, Downey JE, Wang W, Tyler-Kabara EC, Weber DJ, (2013). High-performance neuroprosthetic control by an individual with tetraplegia. The Lancet.

[56] Hochberg LR, Bacher D, Jarosiewicz B, Masse NY, Simeral JD, Vogel J, (2012). Reach and grasp by people with tetraplegia using a neurally controlled robotic arm. Nature.

[57] Lebedev MA, Tate AJ, Hanson TL, Li Z, O’Doherty JE, Winans JA, (2011). Future developments in brain-machine interface research. Clinics.

[58] Cavanagh PR, Komi PV (1979). Electromechanical delay in human skeletal muscle under concentric and eccentric contractions. Eur J Appl Physiol Occup Physiol.

[59] Huang H, Zhang F, Hargrove LJ, Dou Z, Rogers DR, Englehart KB (2011). Continuous locomotion-mode identification for prosthetic legs based on neuromuscular mechanical fusion. Biomed Eng IEEE Trans.

[60] Fleischer C, Hommel G (2008). A human–exoskeleton interface utilizing electromyography. Robot IEEE Trans.

[61] Hoover CD, Fulk GD, Fite KB (2012). The design and initial experimental validation of an active myoelectric transfemoral prosthesis. J Med Devices.

[62] Young AJ, Simon A, Hargrove LJ (2013). An intent recognition strategy for transfemoral amputee ambulation across different locomotion modes. Engineering in medicine and biology society (EMBC), 2013 35th annual international conference of the IEEE.

[63] Donath M (1974). Proportional EMG control for above-knee prosthesis.

[64] Souza JM, Fey NP, Cheesborough JE, Agnew SP, Hargrove LJ, Dumanian GA (2014). Advances in transfemoral amputee rehabilitation: early experience with targeted muscle reinnervation. Curr Surg Rep.

[65] Dawley JA, Fite KB, Fulk GD (2013). EMG control of a bionic knee prosthesis: Exploiting muscle co-contractions for improved locomotor function. Rehabilitation Robotics (ICORR), 2013 IEEE International Conference On.

[66] Au SK, Bonato P, Herr H (2005). An EMG-position controlled system for an active ankle-foot prosthesis: an initial experimental study. Rehabilitation robotics, 2005. ICORR 2005. 9th international conference on.

[67] Hargrove LJ, Simon AM, Young AJ, Lipschutz RD, Finucane SB, Smith DG, (2013). Robotic leg control with EMG decoding in an amputee with nerve transfers. N Engl J Med.

[68] Orizio C (1993). Muscle sound: bases for the introduction of a mechanomyographic signal in muscle studies. Crit Rev Biomed Eng.

[69] Perry-Rana SR, Housh TJ, Johnson GO, Bull AJ, Berning JM, Cramer JT (2002). MMG and EMG responses during fatiguing isokinetic muscle contractions at different velocities. Muscle Nerve.

[70] Yamamoto K, Hyodo K, Ishii M, Matsuo T (2002). Development of power assisting suit for assisting nurse labor. JSME Int J Series C.

[71] Stančin S, Dordević S (2011). MC sensor–a novel method for measurement of muscle tension. Sensors.

[72] Kong K, Jeon D (2006). Design and control of an exoskeleton for the elderly and patients. Mechatronics IEEE/ASME Trans.

[73] Lukowicz P, Hanser F, Szubski C, Schobersberger W, Fishkin KP, Schiele B, Nixon P, Quigley A (2006). Detecting and Interpreting Muscle Activity with Wearable Force Sensors. Pervasive computing. Lecture notes in computer science vol. 3968.

[74] Shull PB, Jirattigalachote W, Hunt MA, Cutkosky MR, Delp SL (2014). Quantified self and human movement: a review on the clinical impact of wearable sensing and feedback for gait analysis and intervention. Gait Posture.

[75] Abdul Razak AH, Zayegh A, Begg RK, Wahab Y (2012). Foot plantar pressure measurement system: a review. Sensors.

[76] Otto Bock US Healthcare (2014). Genium and X3 microprocessor knee online training.

[77] REX Bionics Plc: *REX Personal™ Product Information*. Online. 2014. . Accessed 30 May 2014 http://www.rexbionics.com

[78] Farris RJ, Quintero HA, Goldfarb M (2011). Preliminary evaluation of a powered lower limb orthosis to aid walking in paraplegic individuals. Neural Syst Rehabil Eng IEEE Trans.

[79] Duvinage M, Castermans T, Dutoit T (2011). Control of a lower limb active prosthesis with eye movement sequences. Computational Intelligence, Cognitive Algorithms, Mind, and Brain (CCMB), 2011 IEEE Symposium On.

[80] Fondation Suisse pour les Tèléthèses (FST): *Computer-Wheelchair Interface (CWI)*. Online. 2014. . Accessed 30 May 2014 http://www.fstlab.ch/

[81] Bach-y-Rita P, Kercel SW (2003). Sensory Substitution and the human-machine interface. Trends Cognitive Sci.

[82] Meek SG, Jacobsen SC, Goulding PP (1989). Extended physiologic taction: design and evaluation of a proportional force feedback system. J Rehabil Res Dev.

[83] Raspopovic S, Capogrosso M, Petrini FM, Bonizzato M, Rigosa J, Di Pino G, (2014). Restoring natural sensory feedback in real-time bidirectional hand prostheses. Sci Transl Med.

[84] Clippinger FW, Seaber AV, McElhaney JH, Harrelson JM, Maxwell GM (1982). Afferent Sensory Feedback for Lower Extremity Prosthesis. Clin Orthop Relat Res.

[85] Giggins OM, Persson UM, Caulfield B (2013). Biofeedback in rehabilitation. J Neuroeng Rehabil.

[86] Zambarbieri D, Schmid M, Magnaghi M, Vermi G, Macellari V, Fadda A (1998). Biofeedback techniques for rehabilitation of the lower-limb prosthetic subject. Proc. VII Medicon.

[87] Zambarbieri D, Schmid M, Verni G (2001). Sensory feedback for lower limb prostheses.

[88] Redd CB, Bamberg SJM (2011). A wireless sensory feedback system for real-time gait modification. Engineering in medicine and biology society, EMBC, 2011 annual international conference of the IEEE.

[89] Sigrist R, Rauter G, Riener R, Wolf P (2013). Augmented visual, auditory, haptic, and multimodal feedback in motor learning: a review. Psychon Bull Rev.

[90] Bamberg SJM, Carson RJ, Stoddard G, Dyer PS, Webster JB (2010). The lower extremity ambulation feedback system for analysis of gait asymmetries: preliminary design and validation results. J Prosthet Orthot.

[91] Yang L, Dyer PS, Carson RJ, Webster JB, Foreman KB, Bamberg SJM (2012). Utilization of a lower extremity ambulatory feedback system to reduce gait asymmetry in transtibial amputation gait. Gait Posture.

[92] Gilbert JA, Maxwell GM, George Jr RT, McElhaney JH (1982). Technical note - auditory feedback of knee angle for amputees. Prosthet Orthot Int.

[93] Kaczmarek K, Webster J, Bach-y-Rita P, Tompkins W (1991). Electrotactile and vibrotactile displays for sensory substitution systems. IEEE Trans Biomed Eng.

[94] Kawamura J, Sueda O, Harada K, Nishihara K, Isobe S (1981). Sensory feedback systems for the lower-limb prosthesis. J Osaka Rosai Hospital.

[95] Sabolich JA, Ortega GM (1994). Sense of feel for lower-limb amputees: a phase-one study. J Prosthet Orthot.

[96] Webb G, Ewins D, Ghoussayni S (2012). Electro-tactile sensation thresholds for an amputee gait-retraining system. 3rd annual conference of the international functional electrical stimulation society.

[97] Izumi T, Hoshimiya N (1988). A presentation method of a traveling image for the sensory feedback for control of the paralyzed upper extremity. Syst Comput Japan.

[98] Seps M, Dermitzakis K, Hernandez-Arieta A (2011). Study on lower back electrotactile stimulation characteristics for prosthetic sensory feedback. Intelligent robots and systems (IROS), 2011 IEEE/RSJ international conference on.

[99] Lee M-Y, Soon K-S (2009). New computer protocol with subsensory stimulation and visual/auditory biofeedback for balance assessment in amputees. Systems, man and cybernetics, 2009. SMC, 2009. IEEE international conference on.

[100] Webb G (2011). Providing real-time biofeedback for amputee gait retraining using labview. NI days worldwide graphical system design conference.

[101] Stepp CE, An Q, Matsuoka Y (2012). Repeated training with augmentative vibrotactile feedback increases object manipulation performance. PloS ONE.

[102] Rusaw D, Hagberg K, Nolan L, Ramstrand N (2012). Can vibratory feedback be used to improve postural stability in persons with transtibial limb loss?. J Rehabil Res Dev.

[103] Fan RE, Culjat MO, Kim C-H, Franco ML, Boryk R, Bisley JW, (2008). A haptic feedback system for lower-limb prostheses. IEEE Trans Neural Syst Rehabil Eng.

[104] Pylatiuk C, Kargov A (2006). Schulz S, Design and evaluation of a low-cost force feedback system for myoelectric prosthetic hands. J Prosthet Orthot.

[105] Blank A, Okamura AM (2010). Kuchenbecker KJ, Identifying the role of proprioception in upper-limb prosthesis control: Studies on targeted motion. ACM Trans Appl Perception (TAP).

[106] Pagel A, Oes J, Pfeifer S, Riener R, Vallery H (2013). Künstliches feedback für oberschenkelamputierte–theoretische analyse/artificial feedback for transfemoral amputees–theoretical analysis. at-Automatisierungstechnik.

[107] Sup F, Varol HA (2011). Goldfarb M, Upslope walking with a powered knee and ankle prosthesis: Initial results with an amputee subject. Neural Syst Rehabil Eng IEEE Trans.

[108] Lawson BE, Varol HA (2011). Goldfarb M, Standing stability enhancement with an intelligent powered transfemoral prosthesis. Biomed Eng IEEE Trans.

[109] Li YD, Hsiao-Wecksler ET (2013). Gait mode recognition and control for a portable-powered ankle-foot orthosis. Rehabilitation robotics (ICORR), 2013 IEEE international conference on.

[110] Li Q, Young M, Naing V, Donelan JM (2009). Walking speed and slope estimation using shank-mounted inertial measurement units. Rehabilitation robotics, 2009. ICORR 2009. IEEE international conference on.

[111] Farrell MT (2013). Pattern classification of terrain during amputee walking.

[112] Scandaroli GG, Araujo Borges G, Ishihara JY, Terra MH, da Rocha AF, de Oliveira Nascimento FA (2009). Estimation of foot orientation with respect to ground for an above knee robotic prosthesis. Intelligent robots and systems, 2009. IROS 2009. IEEE/RSJ international conference on.

[113] Zhang F, Fang Z, Liu M, Huang H (2011). Preliminary design of a terrain recognition system. Engineering in medicine and biology society, EMBC, 2011 annual international conference of the IEEE.

[114] Grimes DL (1979). An active multimode above knee prosthesis controller.

[115] Peeraer L, Aeyels B, der Perre GV (1990). Development of emg-based mode and intent recognition algorithms for a computer-controlled above-knee prosthesis. J Biomed Eng.

[116] Popovic D, Tomovic R, Tepavac D, Schwirtlich L (1991). Control aspects of active above-knee prosthesis. Int J Man-Mach Stud.

[117] Quintero HA, Farris RJ, Hartigan C, Clesson I, Goldfarb M (2011). A powered lower limb orthosis for providing legged mobility in paraplegic individuals. Topics Spinal Cord Injury Rehabil.

[118] Zhang F, Liu M, Huang H (2014). Effects of locomotion mode recognition errors on volitional control of powered above-knee prostheses. Neural Syst Rehabil Eng IEEE Trans.

[119] Young AJ, Simon AM, Hargrove LJ (2014). A training method for locomotion mode prediction using powered lower limb prostheses. Neural Syst Rehabil Eng IEEE Trans.

[120] Simon AM, Fey NP, Ingraham KA, Young A, Hargrove LJ (2013). Powered prosthesis control during walking, sitting, standing, and non-weight bearing activities using neural and mechanical inputs. Neural Engineering (NER), 2013 6th International IEEE/EMBS Conference On.

[121] Zhang F, Liu M, Huang H (2012). Preliminary study of the effect of user intent recognition errors on volitional control of powered lower limb prostheses. Engineering in medicine and biology society (EMBC), 2012 annual international conference of the IEEE.

[122] Novak D, Riener R (2014). A survey of sensory fusion methods in wearable robotics. Robot Autonomous Syst.

[123] Kamnik R, Vitiello N, Lefeber D, Pasquini G,, Goršič M (2014). Online phase detection using wearable sensors for walking with a robotic prosthesis. Sensors.

[124] Kawamoto H, Kanbe S, Sankai Y (2003). Power assist method for HAL-3 estimating operator’s intention based on motion information. Robot and human interactive communication, 2003. proceedings. ROMAN 2003. The 12th IEEE international workshop on.

[125] Jin D, Yang J, Zhang R, Wang R, Zhang J (2006). Terrain identification for prosthetic knees based on electromyographic signal features. Tsinghua Sci Technol.

[126] Novak D, Reberšek P, Rossi SMMD, Donati M, Podobnik J, Beravs T, (2013). Automated detection of gait initiation and termination using wearable sensors. Med Eng Phys.

[127] Young AJ, Simon AM, Fey NP, Hargrove LJ (2013). Classifying the intent of novel users during human locomotion using powered lower limb prostheses. Neural engineering (NER), 2013 6th international IEEE/EMBS conference on.

[128] Kuiken TA, Li G, Lock BA, Lipschutz RD, Miller LA, Stubblefield KA, (2009). Targeted muscle reinnervation for real-time myoelectric control of multifunction artificial arms. JAMA.

[129] Huang H, Kuiken TA, Lipschutz RD (2009). A strategy for identifying locomotion modes using surface electromyography. Biomed Eng IEEE Trans.

[130] Hargrove L, Simon A, Lipschutz R, Finucane S, Kuiken T (2013). Non-weight-bearing neural control of a powered transfemoral prosthesis. J Neuroeng Rehabil.

[131] Tkach DC, Hargrove LJ (2013). Neuromechanical sensor fusion yields highest accuracies in predicting ambulation mode transitions for trans-tibial amputees. Engineering in medicine and biology society (EMBC), 2013 35th annual international conference of the IEEE.

[132] Ha KH, Varol HA, Goldfarb M (2011). Volitional control of a prosthetic knee using surface electromyography. Biomed Eng IEEE Trans.

[133] Young AJ, Simon AM, Fey NP, Hargrove LJ (2014). Intent Recognition in a Powered Lower Limb Prosthesis Using Time History Information. Ann Biomed Eng.

[134] Au S, Berniker M, Herr H (2008). Powered ankle-foot prosthesis to assist level-ground and stair-descent gaits. Neural Netw.

[135] Gancet J, Ilzkovitz M, Cheron G, Ivanenko Y, van der Kooij H, van der Helm F,, Workshop EA (2011). MINDWALKER: A brain controlled lower limbs exoskeleton for rehabilitation. Potential applications to space. 11th Symposium on advanced space technologies in robotics and automation.

[136] Össur (2014). POWER KNEE™ Technical Manual.

[137] Shultz A, Lawson B, Goldfarb M (2014). Running with a powered knee and ankle prosthesis. Neural Syst Rehabil Eng IEEE Trans.

[138] Lawson B, Varol HA, Huff A, Erdemir E, Goldfarb M (2013). Control of stair ascent and descent with a powered transfemoral prosthesis. Neural Syst Rehabil Eng IEEE Trans.

[139] Sankai Y, Kaneko M, Nakamura Y (2011). HAL: Hybrid Assistive Limb based on Cybernics. Robotics research. Springer tracts in advanced robotics. vol. 66.

[140] Ekso Bionics: **Ekso Bionics Ekso™ Product Information.** Online. 2014. . Accessed 15 May 2014 http://www.eksobionics.com/

[141] Strickland E (2012). Good-bye, wheelchair. Spectrum IEEE.

[142] Goffer A: **Enhanced safety of gait in powered exoskeletons.** Dynamic walking conference abstract - available online. Argo Medical Technologies, ReWalk 2014. http://dynamicwalking.org/ocs/index.php/dw2014/dw2014/paper/viewFile/17/10

[143] Farris RJ, Quintero HA, Goldfarb M (2012). Performance evaluation of a lower limb exoskeleton for stair ascent and descent with paraplegia. Engineering in medicine and biology society (EMBC), 2012 annual international conference of the IEEE.

[144] Gancet J, Ilzkovitz M, Motard E, Nevatia Y, Letier P, de Weerdt D, (2012). MINDWALKER: Going one step further with assistive lower limbs exoskeleton for SCI condition subjects. Biomedical robotics and biomechatronics (BioRob), 2012 4th IEEE RAS EMBS international conference on.

[145] Duvinage M, Castermans T, Jimenez-Fabian R, Hoellinger T, De Saedeleer C, Petieau M, (2012). A five-state P300-based foot lifter orthosis: Proof of concept. Biosignals and biorobotics conference (BRC), 2012 ISSNIP.

[146] Tkach DC, Lipschutz RD, Finucane SB, Hargrove LJ (2013). Myoelectric neural interface enables accurate control of a virtual multiple degree-of-freedom foot-ankle prosthesis. Rehabilitation robotics (ICORR), 2013 IEEE international conference on.

[147] Baiden D, Ivlev O (2013). Human-robot-interaction control for orthoses with pneumatic soft-actuators – concept and initial trails. Rehabilitation robotics (ICORR), 2013 IEEE international conference on.

[148] Hasegawa Y, Jang J, Sankai Y (2009). Cooperative walk control of paraplegia patient and assistive system. Intelligent robots and systems, 2009. IROS 2009. IEEE/RSJ international conference on.

[149] Hoover CD, Fite KB (2011). A configuration dependent muscle model for the myoelectric control of a transfemoral prosthesis. Rehabilitation robotics (ICORR), 2011 IEEE international conference on.

[150] Hoover CD, Fulk GD, Fite KB (2013). Stair ascent with a powered transfemoral prosthesis under direct myoelectric control. Mechatronics IEEE/ASME Trans.

[151] Hargrove LJ, Simon AM, Lipschutz RD, Finucane SB, Kuiken A (2011). Real-time myoelectric control of knee and ankle motions for transfemoral amputees. JAMA.

[152] Kawamoto H, Lee S, Kanbe S, Sankai Y (2003). Power assist method for HAL-3 using EMG-based feedback controller. Systems, man and cybernetics, 2003. IEEE international conference on vol. 2.

[153] Fleischer C, Reinicke C, Hommel G (2005). Predicting the intended motion with EMG signals for an exoskeleton orthosis controller. Intelligent robots and systems, 2005. (IROS 2005). 2005 IEEE/RSJ international conference on.

[154] Karavas N, Ajoudani A, Tsagarakis N, Saglia J, Bicchi A, Caldwell D (2014). Tele-impedance based assistive control for a compliant knee exoskeleton. Robot Autonomous Syst.

[155] Ferris DP, Gordon KE, Sawicki GS, Peethambaran A (2006). An improved powered ankle-foot orthosis using proportional myoelectric control. Gait Posture.

[156] Karavas N, Ajoudani A, Tsagarakis N, Caldwell D (2013). Human-inspired balancing assistance: Application to a knee exoskeleton. Robotics and biomimetics (ROBIO), 2013 IEEE international conference on.

[157] Vallery H, Burgkart R, Hartmann C, Mitternacht J, Riener R, Buss M (2011). Complementary limb motion estimation for the control of active knee prostheses. Biomedizinische Technik/Biomed Eng.

[158] Holgate MA, Bohler AW, Sugar TG (2008). Control algorithms for ankle robots: a reflection on the state-of-the-art and presentation of two novel algorithms. Biomedical robotics and biomechatronics, 2008. BioRob 2008. 2nd IEEE RAS EMBS international conference on.

[159] Gregg RD, Sensinger JW (2014). Towards biomimetic virtual constraint control of a powered prosthetic leg. Control Syst Technol IEEE Trans.

[160] Asbeck AT, Dyer RJ, Larusson AF, Walsh CJ (2013). Biologically-inspired soft exosuit. Rehabilitation robotics (ICORR), 2013 IEEE international conference on.

[161] Andersen JB, Sinkjaer T (2003). Mobile ankle and knee perturbator. Biomed Eng IEEE Trans.

[162] Sulzer JS, Gordon KE, Hornby TG, Peshkin MA, Patton JL (2009). Adaptation to knee flexion torque during gait. Rehabilitation robotics, 2009. ICORR 2009. IEEE international conference on.

[163] Li D, Becker A, Shorter KA, Bretl T, Hsiao-Wecksler EA (2011). Estimating system state during human walking with a powered ankle-foot orthosis. Mechatronics IEEE/ASME Trans.

[164] Borghese NA, Bianchi L, Lacquaniti F (1996). Kinematic determinants of human locomotion. J Physiol.

[165] Hansen AH, Childress DS (2010). Investigations of roll-over shape: implications for design, alignment, and evaluation of ankle-foot prostheses and orthoses. Disabil Rehabil.

[166] Grimes DL, Flowers WC, Donath M (1977). Feasibility of an active control scheme for A/K prostheses. J Biomed Eng.

[167] Wang WJ, Li J, Li WD, Sun LN (2013). An echo-based gait phase determination method of lower limb prosthesis. Adv Mater Res.

[168] Sreenath K, Park H-W, Poulakakis I, Grizzle JW (2011). A compliant hybrid zero dynamics controller for stable, efficient and fast bipedal walking on MABEL. Int J Robot Res.

[169] Gregg RD, Sensinger JW (2013). Biomimetic virtual constraint control of a transfemoral powered prosthetic leg. American control conference (ACC), 2013.

[170] Gregg RD, Lenzi T, Fey NP, Hargrove LJ, Sensinger JW (2013). Experimental effective shape control of a powered transfemoral prosthesis. Rehabilitation robotics (ICORR), 2013 IEEE international conference on.

[171] Boehler AW, Hollander KW, Sugar TG, Shin D (2008). Design, implementation and test results of a robust control method for a powered ankle foot orthosis (AFO). Robotics and automation, 2008. ICRA 2008. IEEE international conference on.

[172] Eilenberg MF, Geyer H, Herr H (2010). Control of a powered ankle-foot prosthesis based on a neuromuscular model. Neural Syst Rehabil Eng IEEE Trans.

[173] Fite K, Mitchell J, Sup F, Goldfarb M (2007). Design and control of an electrically powered knee prosthesis. Rehabilitation robotics, 2007. ICORR 2007. IEEE 10th international conference on.

[174] Lambrecht BGA, Kazerooni H (2009). Design of a semi-active knee prosthesis. Robotics and automation, 2009. ICRA ’09. IEEE international conference on.

[175] Lawson BE, Varol HA, Sup F, Goldfarb M (2010). Stumble detection and classification for an intelligent transfemoral prosthesis. Engineering in medicine and biology society (EMBC), 2010 annual international conference of the IEEE.

[176] Lawson BE, Shultz AH, Goldfarb M (2013). Evaluation of a coordinated control system for a pair of powered transfemoral prostheses. Robotics and automation (ICRA), 2013 IEEE international conference on.

[177] Liu M, Zhang F, Datseris P, Huang H (2013). Improving finite state impedance control of active-transfemoral prosthesis using dempster-shafer based state transition rules. J Intell Robot Syst.

[178] Murray S, Goldfarb M (2012). Towards the use of a lower limb exoskeleton for locomotion assistance in individuals with neuromuscular locomotor deficits. Engineering in medicine and biology society (EMBS), 2012 annual international conference of the IEEE.

[179] Shultz AH, Mitchell JE, Truex D, Lawson BE, Goldfarb M (2013). Preliminary evaluation of a walking controller for a powered ankle prosthesis. Robotics and automation (ICRA), 2013 IEEE international conference on.

[180] Sun J, Voglewede PA (2013). Powered transtibial prosthetic device control system design, implementation, and bench testing. J Med Devices.

[181] Sup F, Varol HA, Mitchell J, Withrow TJ, Goldfarb M (2009). Preliminary evaluations of a self-contained anthropomorphic transfemoral prosthesis. Mechatronics IEEE/ASME Trans.

[182] Sup F, Bohara A, Goldfarb M (2008). Design and control of a powered transfemoral prosthesis. Int J Robot Res.

[183] Sup F, Varol HA, Mitchell J, Withrow T, Goldfarb M (2008). Design and control of an active electrical knee and ankle prosthesis. Biomedical robotics and biomechatronics, 2008. BioRob 2008. 2nd IEEE RAS EMBS international conference on.

[184] Varol HA, Goldfarb M (2007). Decomposition-based control for a powered knee and ankle transfemoral prosthesis. Rehabilitation robotics, 2007. ICORR 2007. IEEE 10th international conference on.

[185] Wang Q, Yuan K, Zhu J, Wang L (2014). Finite-state control of a robotic transtibial prosthesis with motor-driven nonlinear damping behaviors for level ground walking. Advanced motion control (AMC), 2014 IEEE 13th international workshop on.

[186] Zlatnik D, Steiner B, Schweitzer G (2002). Finite-state control of a trans-femoral (TF) prosthesis. Control Syst Technol IEEE Trans.

[187] Simon AM, Ingraham KA, Fey NP, Finucane SB, Lipschutz RD, Young AJ, (2014). Configuring a powered knee and ankle prosthesis for transfemoral amputees within five specific ambulation modes. PloS one.

[188] Aghasadeghi N, Zhao H, Hargrove LJ, Ames AD, Perreault EJ, Bretl T (2013). Learning impedance controller parameters for lower-limb prostheses. Intelligent robots and systems (IROS), 2013 IEEE/RSJ international conference on.

[189] Wang D, Liu M, Zhang F, Huang H (2013). Design of an expert system to automatically calibrate impedance control for powered knee prostheses. Rehabilitation robotics (ICORR), 2013 IEEE international conference on.

[190] Markowitz J, Krishnaswamy P, Eilenberg MF, Endo K, Barnhart C, Herr H (2011). Speed adaptation in a powered transtibial prosthesis controlled with a neuromuscular model. Philos Trans R Soc B: Biol Sci.

[191] Vallery H, van Asseldonk E, Buss M, van der Kooij H (2009). Reference trajectory generation for rehabilitation robots: complementary limb motion estimation. IEEE Trans Neural Syst Rehabil Eng.

[192] Pfeifer S, Vallery H, Riener R, List R, Perreault EJ (2010). Finding Best Predictors for the Control of Transfemoral Prostheses. AUTOMED Fortschritt-Berichte VDI.

[193] Vallery H, Ekkelenkamp R, van der Kooij H, Buss M (2007). Complementary limb motion estimation based on interjoint coordination: experimental evaluation. Proceedings of the IEEE international conference on rehabilitation robotics (ICORR).

[194] Pfeifer S, Vallery H, Hardegger M, Riener R, Perreault EJ (2012). Model-based estimation of knee stiffness. Biomed Eng IEEE Trans.

[195] Pfeifer S (2014). Biomimetic stiffness for transfemoral prostheses.

[196] Huang GT (2004). Wearable robots. Technol Rev.

[197] Flowers WC, Mann RW (1977). An electrohydraulic knee-torque controller for a prosthesis simulator. J Biomech Eng.

[198] Olivier J, Ortlieb A, Bouri M, Bleuler H (2014). Mechanisms for actuated assistive hip orthoses. Robot Autonomous Syst.

[199] Martelli D, Vannetti F, Cortese M, Tropea P, Giovacchini F, Micera S, (2014). The effects on biomechanics of walking and balance recovery in a novel pelvis exoskeleton during zero-torque control. Robotica.

[200] Colgate JE, Hogan N (1988). Robust control of dynamically interacting systems. Int J Control.

[201] Hogan N (1985). Impedance control: an approach to manipulation: part I - theory. J Dynamic Syst Meas Control.

[202] Vallery H, Veneman J, van Asseldonk E, Ekkelenkamp R, Buss M, van Der Kooij H (2008). Compliant actuation of rehabilitation robots. Robot Automation Mag IEEE.

[203] Hogan N (1985). Impedance control: an approach to manipulation: part II - implementation. J Dynamic Syst Meas Control.

[204] Gomi H, Kawato M (1996). Equilibrium-point control hypothesis examined by measured arm stiffness during multijoint movement. Science.

[205] Pfeifer S, Pagel A, Riener R, Vallery H (2014). Actuator with angle-dependent elasticity for biomimetic transfemoral prostheses. Mechatronics IEEE/ASME Trans.

[206] Tucker MR, Moser A, Lambercy O, Sulzer J, Gassert R (2013). Design of a wearable perturbator for human knee impedance estimation during gait. Rehabilitation robotics (ICORR), 2013 IEEE international conference on.

[207] Lee H, Hogan N (2013). Investigation of human ankle mechanical impedance during locomotion using a wearable ankle robot. Robotics and automation (ICRA), 2013 IEEE international conference on.

[208] Rouse EJ, Hargrove LJ, Perreault EJ, Kuiken TA (2014). Estimation of human ankle impedance during the stance phase of walking. Neural Syst Rehabil Eng IEEE Trans.

[209] Shamaei K, Cenciarini M, Adams A, Gregorczyk KN, Schiffman JM, Dollar A (2014). Design and evaluation of a quasi-passive knee exoskeleton for investigation of motor adaptation in lower extremity joints. Biomed Eng IEEE Trans.

[210] Aguirre-Ollinger G, Colgate JE, Peshkin MA, Goswami A (2012). Inertia compensation control of a one-degree-of-freedom exoskeleton for lower-limb assistance: Initial experiments. Neural Syst Rehabil Eng IEEE Trans.

[211] Riener R, Lunenburger L, Jezernik S, Anderschitz M, Colombo G, Dietz V (2005). Patient-cooperative strategies for robot-aided treadmill training: first experimental results. Neural Syst Rehabil Eng IEEE Trans.

[212] Caputo JM, Collins SH (2013). An experimental robotic testbed for accelerated development of ankle prostheses. Robotics and automation (ICRA), 2013 IEEE international conference on.

[213] Geeroms J, Flynn L, Jimenez-Fabian R, Vanderborght B, Lefeber D (2013). Ankle-knee prosthesis with powered ankle and energy transfer for CYBERLEGs *α*-prototype. Rehabilitation robotics (ICORR), 2013 IEEE international conference on.

[214] Kazerooni H, Steger R, Huang L (2006). Hybrid control of the Berkeley lower extremity exoskeleton (BLEEX). Int J Robot Res.

[215] Dietz V (2002). Do human bipeds use quadrupedal coordination?. Trends Neurosci.

[216] Chandrapal M, Chen X, Wang W (2013). Preliminary evaluation of a lower-limb exoskeleton - stair climbing. Advanced intelligent mechatronics (AIM), 2013 IEEE/ASME international conference on.

[217] Olivier J, Bouri M, Ortlieb A, Bleuler H, Clavel R (2013). Development of an assistive motorized hip orthosis: Kinematics analysis and mechanical design. Rehabilitation robotics (ICORR), 2013 IEEE international conference on.

[218] Popovic D, Tomovic R, Schwirtlich L (1989). Hybrid assistive system-the motor neuroprosthesis. Biomed Eng IEEE Trans.

[219] Ha KH, Quintero HA, Farris RJ, Goldfarb M (2012). Enhancing stance phase propulsion during level walking by combinin FES with a powered exoskeleton for persons with Paraplegia. Engineering in medicine and biology society (EMBC), 2012 annual international conference of the IEEE.

[220] Kazerooni H, Racine J-L, Huang L, Steger R (2005). On the control of the Berkeley lower extremity exoskeleton (BLEEX). Robotics and automation, 2005. ICRA 2005. Proceedings of the 2005 IEEE international conference on.

[221] Kazerooni H, Steger R (2005). The Berkeley lower extremity exoskeleton. J Dynamic Syst Meas Control.

[222] Bellman RD, Holgate MA, Sugar TG (2008). SPARKy 3: Design of an active robotic ankle prosthesis with two actuated degrees of freedom using regenerative kinetics. Biomedical robotics and biomechatronics, 2008. BioRob 2008. 2nd IEEE RAS EMBS international conference on.

[223] Vanderborght B, Albu-Schaeffer A, Bicchi A, Burdet E, Caldwell DG, Carloni R, (2013). Variable impedance actuators: a review. Robot Autonomous Syst.

[224] Junius K, Cherelle P, Brackx B, Geeroms J, Schepers T, Vanderborght B, (2013). On the use of adaptable compliant actuators in prosthetics, rehabilitation and assistive robotics. Robot motion and control (RoMoCo), 2013 9th workshop on.

[225] Koganezawa K, Fujimoto H, Kato I (1987). Multifunctional above-knee prosthesis for stairs’ walking. Prosthet Orthot Int.

[226] Unal R, Behrens SM, Carloni R, Hekman EEG, Stramigioli S, Koopman HFJM (2010). Prototype design and realization of an innovative energy efficient transfemoral prosthesis. Biomedical robotics and biomechatronics (BioRob), 2010 3rd IEEE RAS and EMBS international conference on.

[227] Unal R, Klijnstra F, Burkink B, Behrens SM, Hekman EEG, Stramigioli S, (2013). Modeling of WalkMECH: a fully-passive energy-efficient transfemoral prosthesis prototype. Rehabilitation robotics (ICORR), 2013 IEEE international conference on.

[228] Matthys A, Cherelle P, Damme MV, Vanderborght B, Lefeber D (2012). Concept and design of the HEKTA (Harvest Energy from the Knee and Transfer it to the Ankle) transfemoral prosthesis. Biomedical robotics and biomechatronics (BioRob), 2012 4th IEEE RAS EMBS international conference on.

[229] Oymagil AM, Hitt JK, Sugar T, Fleeger J (2007). Control of a regenerative braking powered ankle foot orthosis. Rehabilitation robotics, 2007. ICORR 2007. IEEE 10th international conference on.

[230] Collins SH, Kuo AD (2010). Recycling energy to restore impaired ankle function during human walking. PLoS ONE.

[231] Tucker MR, Fite KB (2010). Mechanical damping with electrical regeneration for a powered transfemoral prosthesis. Advanced intelligent mechatronics (AIM), 2010 IEEE/ASME International Conference on.

[232] Collins SH (2013). What do walking humans want from mechatronics?. Mechatronics (ICM), 2013 IEEE International Conference On.

[233] Ding Y, Galiana I, Asbeck A, Quinlivan B, De Rossi SMM, Walsh C (2014). Multi-joint actuation platform for lower extremity soft exosuits. Robotics and automation (ICRA), 2014 IEEE international conference on.

[234] Sinnet RW, Zhao H, Ames A (2011). Simulating prosthetic devices with human-inspired hybrid control. Intelligent robots and systems (IROS), 2011 IEEE/RSJ international conference on.

[235] Zhao H, Kolathaya S, Ames AD (2014). Quadratic programming and impedance control for transfemoral prosthesis. Robotics and automation (ICRA), 2014 IEEE international conference on.

[236] International Standards Organization (2014). ISO 13482:2014 Robots and Robotic Devices - Safety Requirements for Personal Care Robots.

[237] Roland E, Moriarty B (1990). System safety engineering and management.

[238] Kletz T (2001). HAZOP & HAZAN: notes on the identification and assessment of hazards.

[239] Li YD, Hsiao-Wecksler ET (2013). Gait mode recognition and control for a portable-powered ankle-foot orthosis. Rehabilitation robotics (ICORR), 2013 IEEE international conference on.

[240] Shirota C, Simon AM, Kuiken TA (2014). Trip recovery strategies following perturbations of variable duration. J Biomech.

[241] Össur: **Power knee™ reimbursement guide: the step-by-step guide to a successful claim.** Online training resource for clinicians. . Accessed 28 June 2014 http://www.ossur.com

[242] Zeilig G, Weingarden H, Zwecker M, Dudkiewicz I, Bloch A, Esquenazi A (2012). Safety and tolerance of the ReWalk™ exoskeleton suit for ambulation by people with complete spinal cord injury: A pilot study. J Spinal Cord Med.

[243] Sánchez A, Poignet P, Dombre E, Menciassi A, Dario P (2014). A design framework for surgical robots: Example of the ARAKNES robot controller. Robot Autonomous Syst.

